# Integrative metabolomics and chemometric analysis reveal metabolites associated with antibacterial and antibiofilm activities of celery, Coriander, and parsley against multidrug-resistant uropathogenic *Escherichia coli*

**DOI:** 10.1186/s12906-026-05610-0

**Published:** 2026-10-21

**Authors:** Shymaa I. A. Abdel-dayem, Rania A. El Gedaily, Basma M. Eltanany, Nevine L. Seiffein, Ghada Ali Hani, Sherif M. Afifi, Dalia E. Ali

**Affiliations:** 1 Pharmacognosy Department, Faculty of Pharmacy, Cairo University, Cairo, 11562 Egypt; 2 Department of Pharmaceutical Analytical Chemistry, Faculty of Pharmacy, Cairo University, Cairo, 11562 Egypt; 3 Microbiology and Immunology Department, Faculty of Pharmacy, Pharos University in Alexandria, Canal El Mahmoudia Street, Beside Green Plaza Complex , Alexandria, 21648 Egypt; 4 Alexandria Main University Hospital, Alexandria University, Alexandria, 21526 Egypt; 5 LAQV-REQUIMTE, Laboratory of Bromatology and Hydrology, Faculty of Pharmacy, University of Porto, Rua Jorge de Viterbo Ferreira 228, Porto, 4050-313 Portugal; 6 Pharmacognosy and Natural Products Department, Faculty of Pharmacy, Pharos University in Alexandria, Canal El Mahmoudia Street, Beside Green Plaza Complex , Alexandria, 21648 Egypt

**Keywords:** Antibiofilm, Antibacterial, Apiaceae, Multidrug-resistant *E*.*Coli*, Uropathogenic bacteria, LC-QTOF-MS/MS, Metabolomics, Complementary medicine.

## Abstract

**Background:**

The emergence of multidrug-resistant (MDR) uropathogenic *Escherichia coli* and the increasing prevalence of biofilm-associated infections have created an urgent need for new antimicrobial strategies. Celery (*Apium graveolens* L.), coriander (*Coriandrum sativum* L.), and parsley (*Petroselinum crispum*) are widely consumed herbs with reported medicinal properties; however, the relationship between their metabolite composition and antimicrobial potential remains insufficiently understood. This study investigated whether differences in the metabolite profiles of these Apiaceae herbs are associated with their antibacterial and antibiofilm activities against clinical MDR uropathogenic *E. coli* isolates.

**Methods:**

Methanolic extracts of celery, coriander, and parsley, together with their derived methylene chloride: water (50:50, v/v) fractions, were evaluated for antibacterial and antibiofilm activities against clinical MDR uropathogenic *E. coli*. Untargeted LC-QTOF-MS/MS metabolomics in positive and negative ionization modes was combined with multivariate chemometric analyses to characterize metabolite profiles, discriminate among the three herbs, and identify metabolites associated with the observed biological activities.

**Results:**

A total of 167 metabolites were annotated, including flavonoids, coumarins, phenolic acids, fatty acids, chlorins, and amino acid derivatives. Chemometric analyses clearly differentiated the three Apiaceae species based on their metabolic composition. Coriander exhibited the strongest antibacterial activity, with a minimum inhibitory concentration of 3.75 µg/mL, while all tested extracts demonstrated significant inhibition of biofilm formation. Integration of metabolomic and bioactivity data highlighted hydroxylated fatty acids, quercetin derivatives, coumarins, chlorin-related metabolites, and eucommicin A as candidate metabolites associated with antibacterial and antibiofilm effects.

**Conclusions:**

This study provides the first integrated metabolomics-guided comparison of celery, coriander, and parsley against clinical MDR uropathogenic *E. coli* isolates. By linking metabolite signatures with antibacterial and antibiofilm activities, the findings offer new insight into the phytochemical basis of the biological effects of these edible herbs and identify promising candidates for future bioassay-guided and mechanistic studies aimed at combating antimicrobial resistance and biofilm-associated infections.

**Graphical Abstract:**

**Figure:**
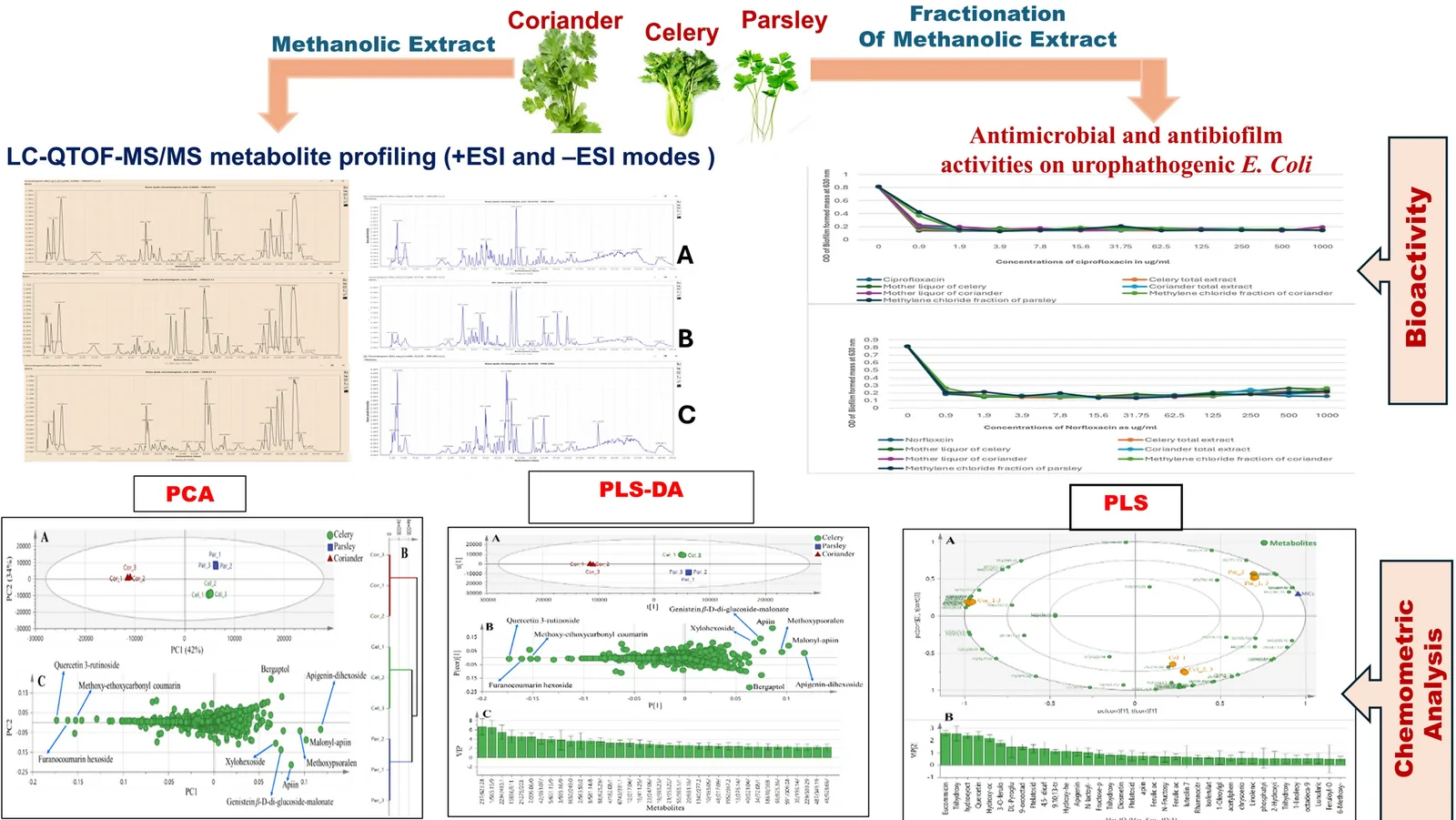

**Supplementary Information:**

The online version contains supplementary material available at https://doi.org/10.1186/s12906-026-05610-0.

## Background

Since ancient times, herbs have been used for their therapeutic and preservative properties. In recent years, growing concern over antimicrobial resistance and the limitations of synthetic preservatives has renewed interest in plant-derived compounds as potential sources of antibacterial and antibiofilm agents. Among these, edible herbs from the Apiaceae family (previously Umbelliferae) [1], such as celery, coriander, and parsley, represent relevant matrices due to their widespread use and chemical diversity.

In many Asian countries, celery (*Apium graveolens* L.) is a common vegetable and flavoring ingredient, largely due to the distinctive aroma of its essential oil [2]. Celery contains a diverse range of secondary metabolites, including flavonoids, alkaloids, terpenoids, phenolic acids, carotenes, tocopherols, and vitamins [3–5]. Previous pharmacological studies have demonstrated that celery exhibits a wide range of bioactivities, including gastrointestinal, cardiovascular, cytotoxic, antibacterial, anthelminthic, hypolipidemic, anti-inflammatory, and neuroactive effects [6].

Coriander (*Coriandrum sativum* L.), an annual herbaceous plant native to the Mediterranean region, is widely cultivated for culinary and medicinal purposes [7]. Phytochemical analyses have revealed the presence of terpenoids, alkaloids, reducing sugars, flavonoids, phenolics, tannins, fatty acids, glycosides, and sterols, in addition to its essential oil [8, 9]. Coriander also contains a wide range of proteins, minerals, trace elements, vitamins, carbohydrates, and dietary fiber, contributing to its high nutritional value. Its extracts have been reported to exhibit anti-inflammatory, analgesic, antibacterial, antioxidant, hypolipidemic, anxiolytic, hypoglycemic, anticancer, and anticonvulsant effects [10, 11].

Parsley (*Petroselinum crispum* (Mill.)) is a biennial aromatic plant rich in vitamin C, vitamin E, carbohydrates, flavonoids, and furanocoumarins [12]. Reported bioactivities of parsley include hypolipidemic [13], antidiabetic, antihypertensive, antibacterial, antioxidant [14], and anticancer effects [15].

Urinary tract infections (UTIs) are among the most common bacterial infections, with *Escherichia coli* (*E. coli*) being the predominant uropathogen. The increasing prevalence of multidrug resistance (MDR) strains, together with their ability to form biofilms or disruption of normal microbiota due to conventional antibiotic treatment, contributes to treatment failure and recurrence [16, 17]. In this context, identifying natural compounds capable of inhibiting bacterial growth and interfering with biofilm formation with minimum side effects is of particular interest.

Liquid chromatography quadrupole time-of-flight tandem mass spectrometry (LC-QTOF-MS/MS)-based untargeted metabolomics has emerged as a powerful tool for the comprehensive characterization of complex plant extracts. Its high separation efficiency, sensitivity, mass accuracy and resolution enable the detection and tentative annotation of a wide range of metabolites in a single analytical workflow [18]. In combination with chemometric approaches, including principal component analysis (PCA), hierarchical cluster analysis (HCA), partial least squares-discriminant analysis (PLS-DA), and orthogonal partial least squares-discriminant analysis (OPLS-DA), LC-QTOF-MS untargeted metabolomics facilitates sample classification and the identification of individual metabolites or metabolite panels associated with specific conditions, including plant origin, organ, processing, and bioactivities [18, 19]. Despite the widespread culinary and medicinal use of celery, coriander, and parsley, limited information is available regarding the relationship between their metabolite composition and their antibacterial and antibiofilm activities against clinically relevant multidrug-resistant (MDR) uropathogenic *E. coli*. We hypothesized that differences in the metabolite profiles of these closely related Apiaceae herbs contribute to variations in their biological activities and that an integrated metabolomics-guided approach could identify metabolites associated with these effects. Celery (*A. graveolens*), coriander (*C. sativum*), and parsley (*P. crispum*) were selected as related edible member of the Apiaceae family with distinct phytochemical profiles and previously reported antimicrobial potential against uropathogenic *E. coli* [20–22]. Their selection therefore provided a rational comparative model to investigate whether interspecies differences in metabolite composition could be linked to differential activity against uropathogenic *E. coli* (UPEC).Therefore, the present study aimed to comprehensively characterize the metabolomes of celery, coriander, and parsley extracts using untargeted LC-QTOF-MS/MS analysis in both positive and negative ionization modes, combined with multivariate chemometric tools for metabolite discrimination and the identification of discriminant metabolite features. In parallel, the antibacterial and antibiofilm activities of the extracts and their derived fractions were evaluated against clinical MDR uropathogenic *E. coli* isolates. By integrating metabolomic and bioactivity datasets, this study established metabolite activity relationships and identified candidate phytochemical markers potentially responsible for the observed biological effects. The novelty of this work lies in the integration of untargeted metabolomics, chemometric analysis, antibacterial screening against clinical MDR uropathogenic isolates, and antibiofilm evaluation within a single analytical framework. This approach not only provides a comparative phytochemical assessment of three commonly consumed Apiaceae herbs but also offers insight into the metabolite signatures associated with their antibacterial and antibiofilm activities, thereby providing a foundation for future bioassay-guided isolation and mechanistic investigations.

## Materials and methods

Fresh plant materials (celery, coriander, and parsley) were purchased from a commercial market (METRO, Alexandria, Egypt) under the trade name “Alandaluis”. The plant samples were taxonomically identified by Prof. Dr. Wafaa M. Amer (Professor of Taxonomy and Flora, Cairo University Herbarium, Faculty of Science, Cairo University). Voucher specimens of celery (A.G.1.6.23), coriander (C.S.1.6.23), and parsley (P.C.1.6.23) were deposited at the Department of Pharmacognosy, Faculty of Pharmacy, Cairo University, Egypt.

### Preparation of plant extracts

Air-dried aerial parts of celery, coriander, and parsley (120 g each) were separately extracted with methanol (3 × 100 mL) using a sonicator SONICA (Soltec Co., Milano, Italy) for 15 min. The extracts were concentrated using a rotary evaporator at 45 °C under reduced pressure to constant weight, yielding viscous residues (23.4, 21.2, and 21.6 g, respectively), and then dried in a desiccator over anhydrous CaCl_2_.

Each methanolic extract was suspended in distilled water (100 mL) and partitioned with methylene chloride (3 × 100 mL) at room temperature to yield methylene chloride fractions (2.8, 1.9, and 2.1 g, respectively) and corresponding aqueous mother liquors (19.6, 18.5, and 17.3 g, respectively). All fractions were concentrated and stored in a desiccator over anhydrous CaCl_2_ until further analysis.

### LC-QTOF-MS/MS analysis

All solvents (water, methanol, and acetonitrile) and chemicals (formic acid) used for LC-QTOF-MS/MS analysis were of analytical or LC-MS grades (Merck, Darmstadt, Germany). Dried methanolic extracts were reconstituted in methanol at 10 mg/mL, followed by centrifugation at 13,250 x g for 10 min at 25 °C and filtration through a 0.22 μm syringe filter. Analyses were performed using an Agilent 1260 Infinity liquid chromatograph coupled to a 6546 LC/QTOF mass spectrometer equipped with an orthogonal ESI interface (Agilent Technologies, Waldbronn, Germany). The QTOF-MS/MS source and acquisition parameters used in the present study are described below, the method was adapted from a previously reported procedure [23]. Briefly, 5 µL of each sample were injected using an autosampler maintained at 4 °C onto a Zorbax SB-C18 column (150 × 2.1 mm, 5 μm particle size). Separation was achieved using a binary gradient of water (A) and acetonitrile (B), both containing 0.1% (v/v) formic acid. Mobile phases degassing performed by sonication for 10 min before use. Chromatographic separation was performed at a flow rate of 0.35 mL/min using the following program: solvent B was initially maintained at 5% for 1 min, increased linearly from 5% to 95% over 15 min, maintained at 95% for 2 min for column cleaning, decreased from 95% to 5% over 2 min. The column was subsequently re-equilibrated at 5% solvent B for 5 min, resulting in a total run time of 25 min. Samples (5 µL) were injected using an autosampler maintained at 4 °C, and chromatographic separation was conducted at room temperature in triplicate. The QTOF mass spectrometer operated under optimized conditions in both positive and negative ESI modes. In negative ESI mode and positive ESI mode, the drying gas flow rate was 8 L/min, the drying gas temperature was 350 °C, the capillary voltage was 3500 V, the nebulizer gas pressure was 30 psi, the skimmer voltage was 60 V, the fragmentor voltage was 150 V, and the OCT 1 RF Vpp voltage was 300 V. Auto-MS/MS acquisition was performed over an *m/z* range of 50–2000, with MS and MS/MS scan rates of 10 and 6 spectra/s, respectively. The collision energy was calculated using the formula (slope × *m/z*)/100 + offset, with an offset of 10 and a slope of 5. Up to nine precursor ions were selected per cycle based on the specified absolute and relative abundance thresholds. Precursor selection was further controlled using a purity cutoff of 30%, a purity stringency of 100%, the isotope model for common organic molecules, and active exclusion, with precursors released after two spectra and 0.5 min. Precursors ions were selected based on their relative abundance, using an isolation window of approximately 1.3 *m/z* was applied. ChemStation LC3D (Agilent Technologies) was used for LC control, while MassHunter (Agilent Technologies) was used for instrument control, data acquisition, and processing of MS/MS data. Raw data files were converted to mzXML format using MSConvert 3.0 (ProteoWizard, https://www.proteowizard.org) [23]. The mzXML files were processed using MZmine 2.53 (https://github.com/mzmine/mzmine2/releases/tag/v2.53) [23]. Molecular features were detected using a centroid algorithm with noise levels set at 1.5 × 10^1^ (positive ESI) and 2 × 10^2^ (negative ESI). All analyses were performed at MS level 1. Chromatograms were constructed and deconvoluted using a local minimum search algorithm, with a peak duration range of 0–0.1 min. For positive ESI mode, the minimum peak height and baseline level were set at 2 × 10^3^ and 1.5 × 10^1^, respectively. These values were 1 × 10^3^ and 2 × 10^2^ for negative ESI. Initial feature detection yielded 4,861 and 3,155 detected LC-MS features in negative and positive ESI modes, respectively. The detected features were subsequently processed through peak detection, chromatographic deconvolution, deisotoping, chromatographic alignment, and filtering to remove redundant or unreliable signals. Following these processing steps, 580 and 512 aligned features were retained in negative and positive ESI modes, respectively, for subsequent metabolite annotation and chemometric analysis. Metabolite annotation was based on retention time (Rt), precursor ion, molecular formula prediction, mass error between accurate and exact monoisotopic molecular masses, and MS/MS fragmentation patterns. Annotation was supported using SIRIUS (https://github.com/bright-giant/sirius/releases/tag/v5.6.3), the KEGG database (https://www.kegg.jp/kegg/compound/), and comparison with previously reported data [23–28]. The final annotated metabolites were identified with a high level of confidence, i.e., level 2: tentatively annotated compounds with library/literature spectral data match [29].

### Microbiological analysis

#### Identification of *E. coli* isolates

The isolates used in the current study comprised twenty uropathogenic *E. coli* isolates obtained from private laboratories across Alexandria, Egypt. The isolates were received as swabs on blood agar (Oxoid Ltd, Basingstoke, UK) and MacConkey agar (Oxoid Ltd)

All isolates were identified based on their phenotypic characteristics. Hemolysin production was also assessed. Isolates were streaked on 5–10% defibrinated human blood agar and incubated for 24 h at 37 ◦C. The presence of a clear zone around the streaks indicated *ß* hemolysis [30].

#### Antimicrobial susceptibility testing

Antimicrobial susceptibility testing was performed using the Kirby-Bauer disk diffusion method [31]. The following antibiotic disks purchased from OXOID were tested against the selected bacterial isolates: amikacin, amoxicillin/clavulanic acid, ampicillin, ampicillin/sulbactam, cefadroxil, cefepime, cefoxitin cefoperazone, cefoperazone/sulbactam, ceftazidime, ceftriaxone, cefuroxime, ciprofloxacin, doxycycline, ertapenem, fosfomycin, gentamicin, imipenem, levofloxacin, meropenem, minocycline, moxifloxacin, ofloxacin, rifampicin, sulfamethoxazole/trimethoprim, tobramycin, sulfamethoxazole/trimethoprim, norfloxacin, nalidixic acid, and nitrofurantoin.

#### Determination of biofilm-forming capacity

The biofilm-forming capacity was evaluated using a modified microtiter plate assay [32]. Overnight cultures of *E. coli* isolates were adjusted to a turbidity equivalent to 0.5 McFarland (10^8^ CFU/mL), then further diluted to 10^6^ CFU/mL (corresponding to approximately 0.2 McFarland). Subsequently, 200 *µ*L of each dilution was aseptically transferred into flat-bottom microtiter plates (Cellstar, Greiner Bio-One, Kremsmünster, Austria) and incubated at 37 °C for 24 h. Tryptone soy broth (TSB) was used as a negative control, and each isolate was tested in triplicate. After incubation, the contents of the wells were aspirated, and the wells were washed three times with 200 *µ*L of sterile saline. Biofilms were fixed with 200 µL of absolute methanol for 15 min. Following fixation, the plates were emptied and air-dried. Crystal violet (200 µL, 2%) was added to each well and incubated for 5 min. The plates were then washed and air-dried. Finally, 160 µL of 33% glacial acetic acid was added to each well, and absorbance was measured at 630 nm using an ELISA plate reader (Biotek, USA).

#### Evaluation of antimicrobial activity of plant extracts alone and in combination with ciprofloxacin and norfloxacin activity

Stock solutions of ciprofloxacin and norfloxacin were prepared by dissolving 400 mg of ciprofloxacin HCl or norfloxacin HCl in 100 mL of sterile distilled water. Plant extracts were dissolved in DMSO to a final concentration of 200 mg/mL and further diluted with 5% DMSO throughout the experiment. Sterile microtiter plates were used, with horizontal wells labeled 1–12 and vertical wells labeled A-G.

##### Part I (MIC determination)

Wells A2-A12 received 100 µL of two-fold serial dilutions of the test antibiotic (ciprofloxacin or norfloxacin) and 100 µL of double-strength broth inoculated with *E. coli* (10^6^ CFU/mL) to determine minimum inhibitory concentration (MIC).

##### Part II (combination assay)

Wells A2-A12 received 50 µL of two-fold serially diluted levofloxacin and 50 µL of sterile distilled water, yielding final concentrations ranging from 1000 to 0.9 µg/mL. Well A1 (100 µL of sterile distilled water) served as the control. To simulate combination therapy for urinary tract infections, wells B1 to B12 received 50 µL of two-fold serial dilutions of the antibiotic under test (ciprofloxacin or norfloxacin) and 50 µL of the extract solution. Wells C-G received the same serial dilutions of the antimicrobial extracts under investigation. Each well (total n = 84) received 100 µL of double-strength nutrient broth inoculated with E. coli (106 CFU/mL). Plates were covered and incubated at 35–37 °C for 14 h. The MIC was defined as the lowest concentration showing no visible turbidity. The combined activity of extracts and antibiotics was calculated using the activity index [33].

$$\:Activity\:index=\frac{MIC\:of\:antibiotic\:in\:combination}{MIC\:of\:antibiotic\:alone}$$

#### Evaluation of antibiofilm activity of plant extracts alone and in combination with ciprofloxacin or norfloxacin

The same checkerboard design described above was applied. After 24 h incubation, plates were washed three times with saline, and biofilms were fixed with 200 µL of 99% methanol for 15 min. The wells were decanted and air dried, stained with 200 µL of 2% crystal violet for 5 min, washed, and dried. Biofilms were then solubilized with 160 µL of 33% glacial acetic acid, and absorbance was measured at 630 nm using an ELISA plate reader. 

#### Detection of virulence genes in *E. coli* isolates

Virulence-associated genes were selected based on their relevance to *E. coli* pathogenicity. These included fimH (fimbrial adhesion associated with UTIs), papC (associated with P fimbriae), sfa/focDE (uropathogenic virulence determinant), afaC (associated with adhesion to colonic mucosa), hlyCA (hemolysin production), and cnf-1 (cytotoxic necrotizing factor 1).

Fresh cultures of selected *E. coli* isolates were grown on blood agar plates. Bacterial DNA was extracted using the boiling method [34], and quantified by measuring the absorbance at 260 nm using a NanoDrop spectrophotometer Thermo Fisher Scientific, Waltham, MA, USA) [35]. PCR reactions were performed in a final volume of 20 μL containing 10 μL of 2 x PCR master mix, 0.5 μL (10 picomol) of each primer, 2 μL DNA template, and PCR-grade water to volume. Amplification was carried out using a Veriti Thermal Cycler (Applied Biosystems, Foster City, CA, USA). Primer sequences and annealing temperatures are listed in Table S2. PCR products were resolved with 1-2% agarose gels prepared in Tris-acetate-EDTA (TAE) buffer, depending on fragment size. Gels were stained with ethidium bromide (2 μg/mL) and visualized under UV illumination [36].

### Chemometric analysis

The aligned feature list obtained from MZmine was normalized prior to multivariate data analysis. PCA, HCA, and PLS-DA were applied to the data matrix [18]. The final feature matrices consisted of nine sample columns corresponding to three plant species analyzed in triplicate, with each row representing an aligned LC-MS feature. A CSV file containing m/z values, retention times, molecular formulas, peak intensities, and metabolite annotations was generated from the processed LC-MS data. The resulting matrices were mean-centered and Pareto-scaled prior to PCA, HCA, and PLS-DA using SIMCA version 14 (Umetrics, Umeå, Sweden) [37]. Pareto scaling enhanced the contribution of low abundance metabolites while minimizing amplification of noise and analytical artifacts frequently observed in metabolomics datasets [38, 39].

## Results and discussion

### LC-QTOF-MS/MS analysis

LC-QTOF-MS/MS was employed to obtain global metabolite profiles of methanolic extracts from celery, coriander, and parsley. Representative total ion chromatograms in both negative and positive ESI modes (-ESI and + ESI) are shown in Figures S1 and S2, and the annotated metabolites are presented in Table S1 according to elution order and chemical class, Rt, accurate mass measurements, molecular formula prediction, MS/MS fragmentation patterns, and comparison with databases and literature enabled the highly confident annotation of 167 metabolites [29]. The annotated metabolites were classified into major classes, including flavonoids and flavonoid glycosides, isoflavones, phenolic acids and phenolic acid glycosides, coumarins, amino acids and derivatives, fatty acids and lipids, chlorins, and lignans.

Among the Apiaceae species investigated, coriander exhibited the richest and most diverse profile of flavonoids and phenolic compounds compared with celery and parsley, highlighting its phytochemical relevance. Quercetin (7) and its glycosides, including quercetin-(acetyl-hexoside) (5), quercetin pentoside (6), quercetin 3-rutinoside (8), quercetin glucuronide (13), quercetin 3-hexoside (15), and quercetin 3,7-dirhamnoside (22), together with apigenin glucuronide (17), luteolin-7-O-glucoside (18), and kaempferol-3-O-glucorhamnoside (23), were exclusively detected in coriander. These flavonoids are widely reported bioactive compounds exhibiting diverse bioactivities, including antioxidant, anticancer, anti-inflammatory, antidiabetic, hepatoprotective anti-Alzheimer’s, antibacterial, and neuroprotective effects [40–47]. In addition, coriander contained numerous phenolic acids and derivatives, such as protocatechuic acid hexoside (45), homovanillyl-O-hexoside (47), 4-O-beta-D-glucosyl-4-coumarate (51), chlorogenic acid butyl ester (54), caffeic acid hexoside (56), feruloyl-O-hexoside (57), vanillic acid (59), 4-O-feruloylquinic acid isomer (63), 5-O-feruloylquinic acid isomer (65), ferulic acid (69), 3,5-dicaffeoylquinic acid (71), and isochlorogenic acid (72), isoferulate (76), which are associated with antimicrobial, anti-inflammatory, antioxidant, antiproliferative, and neuroprotective properties [48, 49]. Coriander also contained several coumarins and isoflavones, including esculetin-O-glucoside (esculin) (78), daphnetin (80), aesculin (81), coriandrone E hexoside (83), methoxy-ethoxycarbonyl coumarin (84), dihydroxyisoflavone (42), and isoprunetin (43), which are known for their antimicrobial, antiviral, antioxidant, anti-inflammatory, anticoagulant, and anticancer activities [50–53].

In contrast, celery was characterized by the presence of luteolin 5-O-glucoside (20), Luteolin-7-O-[β-D-apiofuranosyl(1→2)-(6’’-O-malonyl)]-β-D-glucopyranoside (25), malonylapiin (34), apigenin-O-dihexosyl deoxyhexoside (38), 4,5-dicaffeoylquinic acid (74), and the coumarin marmesin (90), compounds associated with anti-inflammatory, antioxidant, and antidiabetic properties [54].

Parsley, however, contained rhamnetin 3-sophoroside (1), isorhamnetin dihexoside (2), coumaric acid-O-hexoside (49), coniferin (64), coniferol (70), and numerous coumarins such as plumieride (85), coumarin (86), columbianetin (87), ulopterol (88), 6-methoxy-3-acetyl-2 H-chromene (89), cnidilin (98), 5-hydroxyxanthotoxin (99), pinnatin (100), bergaptol (101), and isoimperatorin (102), which exhibit antioxidant, antimicrobial, anticoagulant, antitumor, and vasodilatory activities [48–52]. Collectively, these findings indicate that coriander possesses the highest diversity and abundance of flavonoids and phenolic compounds among the studied species, suggesting notable phytochemical relevance. Celery and parsley, although less diverse, remain valuable complementary sources of specialized metabolites with potential therapeutic interest.

#### Flavonoids and flavonoid glycosides

Flavonoids, widely distributed secondary metabolites in the plant kingdom, have gained significant nutraceutical interest due to their antioxidant, antimicrobial, anti-inflammatory, antitumor, and antimutagenic activities, in addition to their ability to modulate the activity of key enzymes in human cells [55–57].

The flavonoids identified in the three herbs studied were predominantly present in glycosylated forms. Their MS/MS fragmentation patterns revealed characteristic retro-Diels-Alder rearrangements, along with the loss of sugar moieties, H_2_O, and carbonyl groups. A total of twenty-three flavonoids were detected in the celery profile, with apigenin conjugates (9 metabolites) being the dominant type, followed by luteolin conjugates (5 metabolites), in addition to apigenin and diosmetin aglycones. Chrysoeriol 7-*O*-apiosylglucoside (30) molecular ion appeared at m/z 593.1500, corresponding to the molecular formula C_27_H_29_O_15_^−^, and produced fragment ions at m/z 299 [M-162-132] and m/z 255 [Ag-2H-CO] (Fig.S6). Chrysoeriol, a 3’-*O*-methyl derivative of luteolin, also yielded a fragment ion at m/z 284 [Ag-15] following loss of a methyl group [58].

Apigenin-*O*-acetyl-hexosyl-pentoside (35) with molecular ion m/z 605.1502 [M-H]^−^corresponding to C_28_H_30_O_15_ in -ESI mode, gave rise to peak m/z 563 [M-42], and base peak m/z 269 resulting from the loss of CO followed by loss of hexosyl - pentosyl sugar units [M-42-162-132] (Fig.S7) [59]. Coriander profile revealed the abundance of 21 flavonoidal metabolites of flavone type including quercetin, apigenin, diosmetin aglycones and their conjugates in addition to luteolin conjugates. Vicenin (3) the only identified *C*-glycoside was found in coriander only with molecular ion at m/z 593.1499 [M-H]^−^ corresponding to C_27_H_30_O_15_ in -ESI mode, with characteristic fragments for *C*-glucosides with a base peak at m/z 383 [Ag-H + 114], m/z 353 [Ag-H + 84], m/z 325 [Ag-H + 56], and m/z 297 [Ag-H + 28] (Fig.S4) [60]. Quercetin conjugates dominated the coriander profile as it was assigned in more than half of the flavonoidal configuration (12 metabolites). Coriander quercetin conjugates were of *O*-glycosides type with a base peak at m/z 303 or 301 in + ESI mode. Quercetin 3-rutinoside (8) showed a molecular peak at m/z 611.1607 [M + H] ^+^ corresponding to C_27_H_30_O_16_, a base peak at m/z 303[M- rutinoside], other fragment m/z 257 (Fig.S5) [28]. Luteolin-7-*O*-glucoside (18) revealed molecular ion 477.0952 [M-H]^−^ with predicated chemical formula C_21_H_20_O_11_, a predominate base peak ion at m/z 285 as result of loss of hexoside sugar unit [M-162], other fragments m/z 255 [Ag-2 H-CO] and m/z 151 [Ag-H-C_8_H_4_O-H_2_O] resulting from retro-Diels-Alder rearrangement and cleavage of ring A at bond C3-C4 and O1-C2 [61]. Parsley flavonoid profile revealed the abundance of 20 metabolites, including rhamnetin, isorhamnetin, luteolin, and apigenin conjugates in addition to apigenin and diosmetin aglycon. Isorhamnetin dihexoside (**2**) gave molecular ion at m/z 639.1553 [M-H]^−^ corresponding to molecular formula C_28_H_32_O_17_. A characteristic peak at m/z 477 as result of loss of hexoside sugar unit [M-162] followed by a loss of another hexoside sugar unit producing a base peak at m/z 315[M-H-162-162] (Fig.S3) [28]. Apigenin glucuronide (17) eluted a molecular ion at m/z 449.1071 corresponding to molecular formula (C_21_H_20_O_11_) in + ESI mode, showed a prominent base peak at m/z 271 as result of loss of glucuronide unit [M-178] [28].

#### Phenolics and phenolic glycosides

Upon the analysis of the phenolic chemical profile of the three herbs under investigation, celery revealed the presence of 11 phenolic metabolites, while coriander and parsley showed 28 and 10 identified phenolic compounds, respectively. Several phenolic glycosides were identified in the analysis, with peak numbers 47, 51, 56, and 57 (Table S1) corresponding to homovanillyl-O-hexoside, 4-O-beta-D-glucosyl-4-coumarate, caffeic acid hexoside, and feruloyl-O-hexoside, respectively. The molecular ions [M-H]⁻ for these compounds were observed at m/z 343.1025, 325.0919, 341.1231, and 355.1024, corresponding to the molecular formulas (C₁₅H₁₉O₉)⁻, (C₁₅H₁₇O₈)⁻, (C₁₆H₂₁O₈)⁻, and (C₁₆H₁₉O₉)⁻. A key diagnostic feature was the presence of daughter ions resulting from the loss of a hexosyl sugar unit [M-162], yielding fragments at m/z 181 (Fig.S8), 163 (Fig.S10), 179 (Fig.S12), and 193 (Fig. S13), respectively [49].

Caffeoylquinic acid (50 and 53) molecular ion was detected in both -ESI and + ESI modes at m/z 355.1021 [M + H] ⁺ and 353.0870 [M-H] ⁻, respectively, corresponding to the molecular formula C_16_H_18_O_9_. The rise of daughter ion at *m/z* 163 [M-192] resulting from loss of quinic acid moiety and other fragments at *m/z* 145, 135, and 117, was observed (Fig.S9) [58]. Chlorogenic acid (C_16_H_18_O_9_) (55) and 3-*O*-feruloylquinic acid (C_17_H_20_O_9_) (60) eluted molecular ion m/z 353.0871 [M-H]^−^ and 367.1025 [M-H]^−^, respectively, revealed similar fragmentation pattern with the rise of daughter ions m/z 191 corresponding to [quinic acid-H], *m/z* 173 resulting from loss of H_2_O molecule [quinic acid-H-18] (Fig. S11 and S14) [62]. 3,5- Dicaffeolquinic acid (C_25_H_24_O_12_) (71), eluted molecular ion at *m/z* 515.1183 [M-H]^−^. Fragmentation through the loss of two neutral caffeic acid moieties resulted in a daughter ion at *m/z* 191 [M-H-2 caffeoyl]. A fragment at *m/z* 135 was due to loss of CO_2_ from caffeic acid (Fig.S15) [63].

#### Furanochromones

Three furanochromones were identified in celery and two in coriander, while the metabolic profile of parsley elevated the presence of eleven, highlighting a distinct difference. Oxypeucedanin hydrate (91) was detected with molecular peak at m/z 305.1021 corresponding to C_16_H_17_O_6_^+^,and was fragmented to give a peak ions at m/z 203 [M-C_5_H_10_O_2_] and m/z 175 [M-C_5_H_10_O_2_-CO] (Fig. S17) [64]. Isopimpinellin (93) elevated a molecular ion peak at m/z 247.0601 [M + H]^+^ corresponding to molecular formula C_13_H_11_O_5_^+^ gave rise to the following fragmentation pattern m/z 217 due to loss of two methyl groups [M-CH_3_-CH_2_], m/z 232 [M-CH_3_], and m/z 189 [M- 2CH_3_-CO] (Fig. S16) [65]. Oxypeucedanin (96) eluted a molecular ion at m/z 287.0912 in + ESI mode, molecular formula C_16_H_15_O_5_^+^ with daughter ions at m/z 203 [M-C_5_H_8_O] and 175 [M-C_5_H_8_O-CO] (Fig. S18). Phellopterin (97), m/z 301.1070 [M + H]^+^ C_17_H_17_O_5_^+^, was fragmented due to loss of isobutenyl moiety followed by CO loss forming ions at m/z 233 and m/z 203, respectively [66]. Isoimperatorin (102), m/z 271.0965 [M + H]^+^ corresponding to C_16_H_15_O_4_^+^, revealed a base peak resulting from the loss of isoprene unit at m/z 203 [M + H-C_5_H_8_ ], while other ions resulted from successive loss of 2CO followed by one molecule of CO_2_ were detected at m/z 175 [M + H-C_5_H_8_-CO], m/z 147 [M H- C_5_H_8_-2CO], and m/z 131 [M + H-C_5_H_8_CO_2_-CO], respectively (Fig.S19) [67].

#### Fatty acids and lipids

The provided lipid-related compounds (Table S1) can be classified into several main groups, including hydroxylated fatty acids, fatty acid amides, glycerophospholipids, and sulfonic acid derivatives. Hydroxylated fatty acids, including three isomers of trihydroxy-octadecaenoic acid (Peaks 121, 124, and 125; [M-H]^−^ at m/z 329.2328), dihydroxy- and hydroxy-octadecatrienoic acids (Peaks 130 and 136; [M-H]^−^ at m/z 309.2064 and 293.1783, respectively), and hydroxy-hexadecanoic acid (147, [M-H]^−^ at 271.2274). According to established literature, the fragmentations of such compounds are diagnostically characterized by cleavage, which occurs at the bond adjacent to the hydroxyl. This cleavage is typically accompanied by neutral losses of H_2_O, CO and CO_2_ [68]. The fatty acid amides are represented by oleamide (151, [M + H]^+^ at m/z 282.2800), palmitamide (152, [M + H]^+^ at m/z 256.2635), and docosenamide (153, [M + H]^+^ at m/z 338.3416), The fragmentation of fatty acid amides is primarily governed by *β*-hydrogen rearrangement near the amide group, protonation causes a hydrogen from the *β*-carbon to migrate toward the carbonyl oxygen, weakening the adjacent C- C bond and initiates the cleavage of the acyl chain. This process generates a homologous series of charged ions that retain the amide moiety, differing by CH2 units [69]. A constant diagnostic fragment at m/z 57 appears in all primary fatty acid amides due to a small acyl or alkyl cation formed by tail rearrangement. In monounsaturated amides like oleamide, specific bond cleavages yield additional characteristic ions at m/z135 (C_10_H_15_) and m/z 121 (C_9_H_13_) arise from fragmentation near the double bond (cleavage before and at the double bond, respectively) [69]. Peaks at m/z 83 and 69 originate from charge-directed fragmentation of the alkyl carbocation produced after the loss of ammonia and water from the carboxamide [69]. A group of glycerophospholipids was identified, including octadecadienoyl-glycero-3-phospho-myoinositol (128, [M-H]^−^ at 595.2877), 1-oleoylglycerophosphoinositol (131, at [M-H]^−^ 597.3035), and 1-linoleoyl–glycero-3-phosphoethanolamine (137, [M-H]^−^ at 476.2773). A common fragmentation pathway involves the loss of their respective head groups glycerolphospho-myoinositol, glycerophosphoinositol, and glycerolphosphoethanolamine which consistently gives rise to a characteristic fragment peak at m/z 279. This ion corresponds to the deprotonated fatty acid moiety [70]. The analysis further revealed several sulfonic acid derivatives, including undecylbenzenesulphonic acid (135, [M-H]^−^ at m/z 311.1679), dodecylbenzenesulfonate (141, [M-H]^−^ at m/z 325.1835), and ethylundecyl benzene sulfonic acid (145, [M-H]^−^ at 339.1990). The common diagnostic feature across the fragmentation of these compounds was the presence of a base peak at m/z 183, which corresponds to the benzenesulphonate ion, confirming the presence of the sulphonic acid head group [71–74].

#### Chlorins

About 12 compounds were identified in + ESI mode in celery, coriander, and parsley, namely pheophytin-B (C_55_H_72_N_4_O_6_) (155), 15^1^ -lactone-pheophorbide A (C_36_H_40_N_4_O_6_) (156), bacteriochlorin A (C_34_H_38_N_4_O_7_+Na) (157), 13^2^-hydroxy-pheophorbide A (C_35_H_36_N_4_O_6_) (158), 13^2^-hydroxy-pheophytin-B (C_55_H_72_N_4_O_7_) (160), Pheophorbide A (C_35_H_36_N_4_O_5_) (161), 15^2^-methoxylactone pheophorbide-B methyl ester (C_37_H_38_N_4_O_8_) (162), methyl pheophorbide B (C_36_H_36_N_4_O_6_) (163), 15^2^-hydroxylactone pheophorbide-A methyl ester (C_36_H_38_N_4_O_7_) (164), 13^2^-hydroxy-pheophytin A (C_43_H_78_N_6_O_13_) (165), and 13^2^-hydroxypheophorbide-A methyl ester (C_36_H_38_N_4_O_6_) (166). All of them gave rise to similar fragmentation pattern with daughter ions at m/z 593 resulting from the loss of terminal phytadiene chain, followed be the loss of carbomethoxy group to give an ion peak at m/z 533, while other daughter ions were observed at m/z 460 due to loss of carbomethoxy group from C13^2^, and m/z 477 resulting from loss of substituents at C17 (Fig. S19-21) [75].

#### Amino acids and amino acid derivative

The identified amino acids (Table S1) included the essential aromatic amino acids such as phenylalanine (109, [M-H]^−^ m/z 164.0713) and L-tryptophan (110, [M + H] ^+^ m/z 205.0972). These aromatic amino acids shared a common fragmentation pathway: an initial loss of ammonia (-17), yielding fragments at m/z 147and 188 respectively, by the subsequent loss of CH_2_CO to produce fragment ions at m/z 103 and 146 respectively [76]. The modified and acetylated amino acids including N-lactoyl-phenylalanine (107, [M-H]^−^ m/z 236.0921), and acetyl phenylalanine (115, [M-H]^−^ m/z 206.0817), fragmented by the loss of lactoyl and acetyl moieties to give m/z 164 corresponding to phenylalanine [76]. The list of compounds also contains amino acid sugar conjugates, including N-fructosyl pyroglutamate (105, [M-H]^−^ m/z 290.0875) and fructose-phenylalanine (108, [M-H]^−^ m/z 326.1238). Fragmentation of these compounds consistently involved the loss of the fructosyl moiety (-162), resulting in product ions at m/z 128 and 164, respectively, which correspond to the respective attached amino acid ion [77].

#### Lignan

Eucommicin A (167, [M-H]^−^ m/z 707.1813) a lignan identified in celery and coriander extracts in the negative ionization mode. The compound exhibited a deprotonated molecular ion [M-H]^−^ at 707.1813, with a base peak at m/z 191 corresponding to the quinic acid moiety [78].

### Microbiological analysis

#### Identification of *E. coli* isolates

The twenty uropathogenic *E. coli* isolates were identified based on their phenotypic characteristics. The isolates appeared as bright pink colonies when cultured on MacConkey agar. Fifteen out of the 20 *E. coli* isolates were *β* Hemolytic.in addition to IMVC (Indole, Methyl Red, Voges-Proskauer, and Citrate) test results.

#### Antimicrobial susceptibility testing

**Fig. 1:**
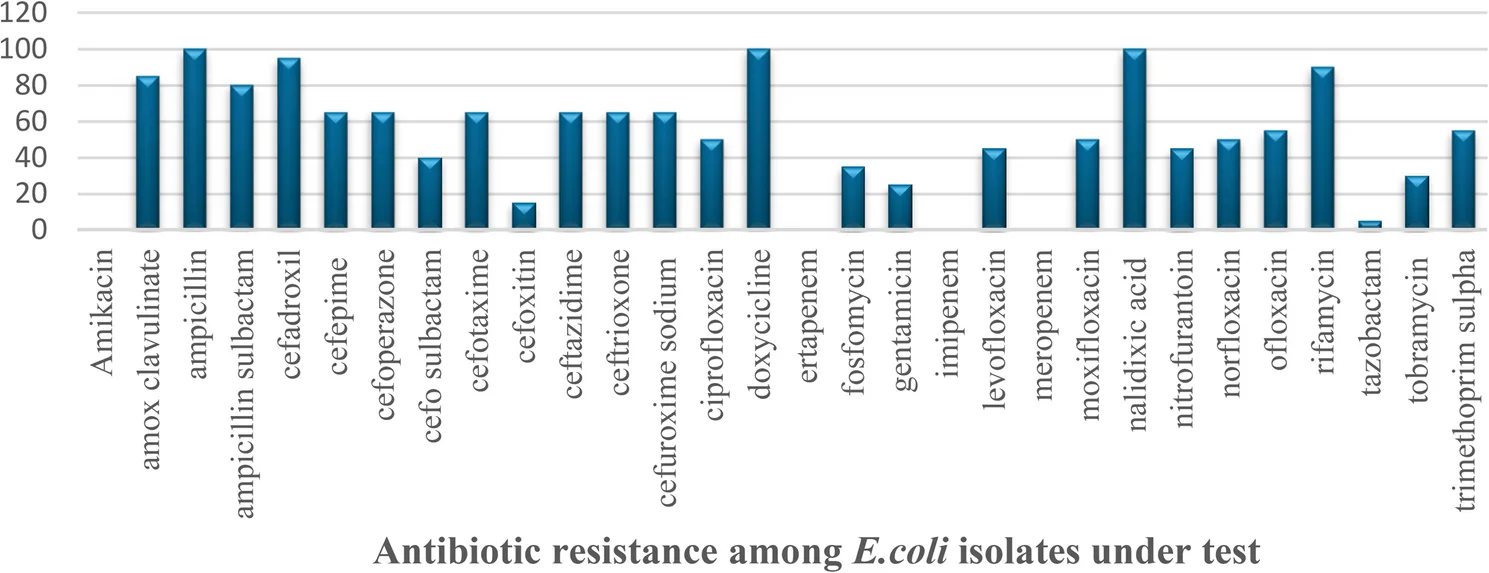
Antibiotic resistance among *E.coli* isolates under test

When Kirby Bauer test was performed the resistance pattern was as follows:

From Fig. 1, based on the interpretation of inhibition zone diameters according to CLSI guidelines, all tested isolates were resistant to Amoxicillin + Clavulanic acid, Doxycycline, and Nalidixic acid. Conversely, they demonstrated 100% sensitivity to Ertapenem, Imipenem, and Meropenem. Given their resistance to multiple antimicrobial categories, most of the isolates were classified as MDR.

#### Determination of biofilm-forming capacity of all isolates

The isolates showed different biofilm-forming capacities, where 12/20 were strong biofilm formers, 4 intermediate biofilm formers, and 4 non-biofilm formers; none of the isolates were weak biofilm formers.

When the selected virulence genes were tested in *E. coli* isolates, their prevalence was as shown in Table 1.

**Table 1:** Prevalence of the tested genes among *E. coli* isolates

Gene	Number	Prevalence
fimH	16/20	80%
Pap	15/20	75%
Hly	15/20	75%
Cnf	7/20	35%
Sfa	11/20	55%
Afa	18/20	90%

In the present study, some of the genes that add to the virulence of uropathogenic *E. coli* were tested. It was found that the prevalence of fimH genes reached 80%. fimH is crucial during UTI for controlling colonization and subsequent invasion of the bladder epithelium and formation of intracellular bacterial communities that could increase bacterial attachment to cells and increased virulence of *E.coli* as reported by Sarshar et al. [79]. The adhesion-encoding genes studied, *pap*, *sfa* and *afa* are prevalent in *E. coli* strains associated with UTIs in humans. As for the *pap* gene its prevalence rose to 76%. The *papC* gene of uropathogenic *E.coli* is crucial for the digalactoside-binding pap pili leading to increased pathogenicity of isolates carrying this gene as noted by Norgren et al. [80].

The *Sfa* gene, which encodes S fimbriae, was detected in about half the *E.coli *isolates. S fimbriae are important virulence factors that contribute to the pathogenicity and virulence of UTI *E.coli* by promoting its adhesion and intracellular activity [81]. The *afa *gene clusters prevalence reached 90% in the current study. These genes are known to encode afimbrial adhesins (Afas), which are expressed by uropathogenic in addition to diarrhea-associated *E. coli* strains, and contribute to adhesion and invasion of the cellular matrix [82]. The *Hly* gene prevalence was 76% among the *E. coli *isolates. It is a highly significant virulence component released by uropathogenic *E.coli.* It is known to be connected to higher UTIs, resulting in pyelonephritis. *E.coli* with α-hemolysin (hlyA) produces large, clear zones of hemolysis around colonies on 5% sheep blood agar [83].* Cnf *genes had the least prevalence among the virulence genes examined, being detected in 35% of the *E. coli *isolates. The *Cnf *genes encodes cytotoxic necrotizing factor 1, a toxin secreted by extra intestinal *E. coli* isolates and had a low prevalence among examined *E. coli* isolates.

#### Evaluation of antimicrobial activity of plant extracts alone or in combination with ciprofloxacin and norfloxacin

As a result of consumer’s negative perception towards synthetic artificial chemicals, natural antimicrobial compounds have gained a great interest [84, 85]. Celery, coriander, and parsley are three herbs that belong to the Apiaceae family and have been used for culinary and medicinal purposes. They contain various phytochemicals such as terpenoids, phenols, flavonoids and essential oils that have antioxidant and antimicrobial properties [1, 2, 8, 9, 12, 86].

The total methanolic extract from the three edible herbs and the two fractions resulting from the subsequent extraction with methylene chloride: water (50:50 v/v) were tested alone against the selected isolates of *E. coli ***(**Table 2**)** associated with UTIs, either alone or in combination with two antibiotics commonly used for UTI treatment, ciprofloxacin and norfloxacin.

Antimicrobial activity varied considerably among the samples. For Celery, the total methanolic extract presented a MIC of 50 *µ*g/mL, while the aqueous mother liquor from subsequent extraction with methylene chloride: water (50:50 v/v) exhibited improved activity (MIC = 25 *µ*g/mL). In contrast, the methylene chloride fraction displayed no detectable activity under the tested conditions. Coriander displayed the highest overall potency, with its total methanolic extract exhibiting an MIC of 3.75 *µ*g/mL. The aqueous mother liquor retained comparable activity (MIC = 3.75 *µ*g/mL), whereas the methylene chloride fraction showed reduced activity (MIC = 100 *µ*g/mL). Finally, Parsley exhibited no detectable activity in either the total methanolic extract or the aqueous mother liquor. However, its methylene chloride fraction showed reduced activity (100 *µ*g/mL).

The observed variability in antimicrobial activity may be attributed to differences in the chemical composition of the extracts and their solubility profiles. In particular, the flavonoid-rich composition of coriander, mainly quercetin, apigenin, and diosmetin aglycones and their conjugates, along with luteolin derivatives and phenolic compounds, has been previously associated with strong antimicrobial effects against *E*.*coli* [87]. These results are consistent with previous reports. For instance, Khalil et al. demonstrated antimicrobial activity of coriander against a range of microorganisms, including *E.coli* [88]. Similarly, Ali et al. reported significant antimicrobial effects of coriander seed and herb extracts against two bacterial strains (*Salmonella typhi* and *Staphylococcus aureus* [11]. Moreover, Kačániová et al. showed that coriander exhibited strong activity against *Stenotrophomonas maltophilia* and *Bacillus subtilis* in both planktonic and biofilm states [89]. This antimicrobial activity was attributed, at least in part, to disruption of the bacterial cytoplasmic membrane, as suggested by H. yildiz, who tested the effects of coriander ethanolic extracts on various bacterial isolates [90].

Regarding ciprofloxacin and norfloxacin, the MICs ranged between 0.9 and 1.9 µg/mL when tested alone. When combined with the extracts investigated, change in MIC values was observed. The combination of coriander methanolic extract with ciprofloxacin and norfloxacin resulted in an additive effect, with no evidence of synergism or antagonism. These findings contrast with those reported by Aelenei et al. [91], who found synergistic interactions between coriander extracts and several antibiotics, including ciprofloxacin, against selected Gram-positive bacteria (methicillin-susceptible and -resistant *Staphylococcus aureus* and *S. epidermidis*) and Gram-negative bacteria (*Pseudomonas aeruginosa* and *E.coli*).

The extracts of celery, coriander and parsley can scavenge free radicals and prevent lipid oxidation, thus protecting against oxidative stress and cellular damage [14, 92–95]. They can also inhibit the growth of various bacterial, fungal and yeast strains, such as *E. coli*,* S. aureus*,* Bacillus cereus*,* Candida albicans*,* Aspergillus flavus* [96]. Several hydroxylated fatty acids (120–122, 124, 125,127, 134 136, and 140) were highlighted in the current study as potential markers to distinguish between celery, coriander and parsley extracts. Hydroxy unsaturated fatty acids had been reported to possess antimicrobial activity related to specific structural attributes [97]. In addition, the unique structure function correlations of fatty acids in the prevention of foodborne spoiling were significantly influenced by the presence of hydroxy groups. The occurrence of hydroxy groups in the central region of the carbon chain is essential for antimicrobial properties of hydroxy unsaturated fatty acids [98]. Derivatives of hydroxy fatty acids had drawn more attention because of their cytotoxic, and anti-inflammatory properties aside from antibacterial attributes [99]. Likewise, several hydroxylated fatty acids i.e., trihydroxy-octadecaenoic acid (124), hydroxy-octadecatrienoic acid (136), and hydroxy-octadecadienoic acid (140) reported herein were enriched in coriander, thus they may contribute to its detected antimicrobial effect.

Moreover, quercetin 3-*O*-rutinoside (8) was abundant in coriander samples in LC-MS dataset from both -ESI and + ESI modes. This specific flavonoid revealed significant antibacterial properties against Gram-negative bacteria i.e., *E. coli* and *Haemophilus influenza* and Gram- positive bacteria i.e., *S. aureus* and *Streptococcus pneunoniae* in a previous study [100]. The antimicrobial activity of celery, coriander and parsley extracts may be attributed to their ability to disrupt the microbial cell membrane, inhibit the microbial enzymes, induce oxidative stress in the microbial cells, or interfere with the microbial quorum sensing [101].

**Table 2:** The Mean of the Minimum inhibitory concentration (MIC) of total methanolic extract from the three edible herbs and the two fractions resulting from the subsequent extraction with methylene chloride: water (50:50 v/v)

No.	Extract	MIC (µg/mL)	Ranges of MIC (µg/mL)
1	Celery total methanolic extract	50	25–75
**2**	Celery aqueous mother liquor	25	12.5–50
**3**	Celery methylene chloride fraction	Inactive under tested conditions	Inactive under tested concentration
4	Coriander total methanolic extract	3.75	1.875-7
**5**	Mother liquor of coriander	3.75	1.875 -7
**6**	Methylene chloride fraction of coriander	100	50–200
7	Parsley total methanolic extract	Inactive under tested concentration	Inactive under tested concentration
**8**	Mother liquor of parsley	Inactive Under tested concentration	Inactive Under tested concentration
**9**	Methylene chloride fraction of parsley	100	50–200

#### Evaluation of biofilm inhibitory activity of plant extracts alone or in combination with ciprofloxacin and norfloxacin

Biofilm formation plays a critical role in the pathogenicity of *E. coli*, as it significantly enhances bacteria’s ability to colonize, persist, and resist host defenses. These biofilms enable bacterial cells to adhere firmly to host tissues or other surfaces, ensuring their long-term survival and causing chronic infections. The biofilm acts as a protective barrier, shielding the bacteria from host immune system and reducing antibiotic penetration, complicating the therapeutic management of infections [102–104]. When the extracts were tested for their ability to inhibit biofilm production, all extracts, alone or in combination, significantly reduced the mass of biofilms formed.

**Fig. 2:**
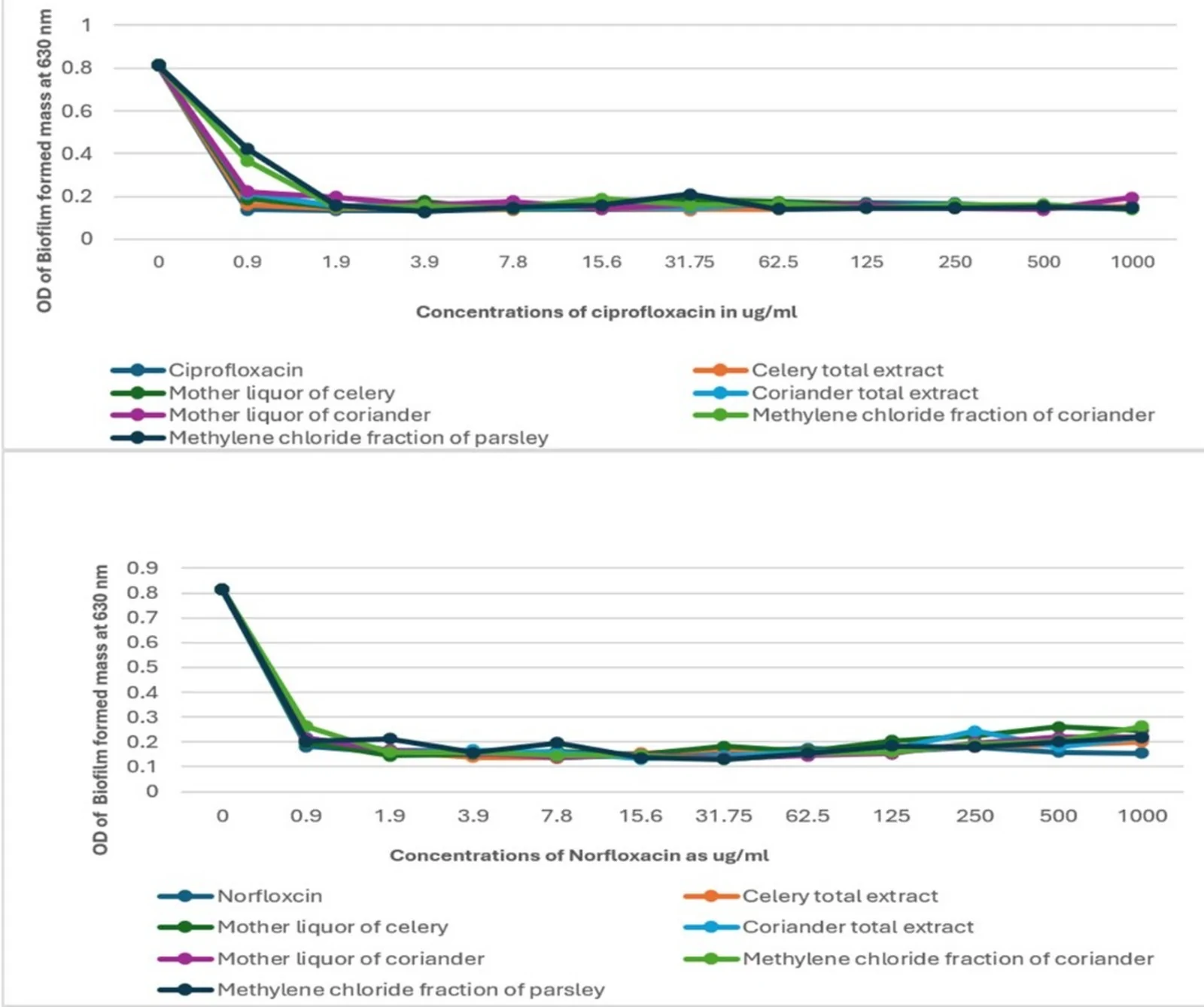
**A **Decrease in biofilm-formed mass by the *E. coli *under test with selected extracts combined with Ciprofloxacin. **B** Decrease in biofilm-formed mass by the *E. coli* under test with selected extracts combined with Norfloxacin

Such behavior is shown in Fig. 2; exemplified by one of the tested *E.coli* strains carrying all the genes under investigation, where the x axis, represent the different concentrations of the antibiotics under test (A) Ciprofloxacin and (B) Norfloxacin, Y axis represent OD of the biofilm reading at 630 nm, the curve shows the values of the effect of the antibiotics alone and in combination with sub MIC values (1/2/MIC) of extracts under test to emphasis their impact on biofilm formed mass.

Plant-derived metabolites, particularly polyphenols and essential oils, act as potent antibiofilm agents by targeting bacterial pathogens such as *P.aeruginosa*,* S. aureus*, and *E. coli* [105–108]. These compounds are found in various herbs like celery, coriander and parsley. A previous study proved that celery oleoresin significantly inhibited biofilm formation of the Gram-negative pathogen *P. aeruginosa* [106]. Coriander possesses significant antibiofilm activity, largely attributed to its essential oils and phenolic compounds, which act through multiple mechanisms. Studies have shown that its methanol and dichloromethane extracts, as well as its essential oil, can effectively inhibit adhesion and biofilm formation in a concentration-dependent manner. This includes pathogenic bacteria such as *E. coli*,* P. aeruginosa*,* Stenotropomonas maltophilia* and *S.aureus*. The mode of action involves interference with quorum sensing (QS) pathways essential for biofilm maturation, suppression of extracellular polymeric substance production (EPS) that stabilizes the biofilm matrix and disruption of bacterial cell wall integrity [107–109]. In addition to essential oils, flavonoids and phenolics are well-documented to be responsible for the antibiofilm activity. As classes of polyphenols, they inhibit biofilm formation through several key mechanisms [110–113]. Quercetin, for example, damages bacterial cell walls, inhibits nucleic acid synthesis, decreases virulence factors production and reduces biofilm formation in Gram-negative bacteria *P. aeruginosa* and *E. coli* [110]. Quercetin-3-glucoside, inhibits biofilm formation in *Bacillus subtilis*, *P. aeruginosa*, *S. aureus*, and *E. coli* [105]. Rutin and apigenin-7-O-glucoside flavonoids inhibit biofilm formation by reducing EPS synthesis and interfering with QS in *S. aureus* and *E. coli* [111, 112]. Other polyphenols, such as luteolin [113], esculin, esculetin, p-coumaric, ferulic acids and psoralen [105], also demonstrate antibiofilm activity against *E. coli*,* P. aeruginosa* and *Enterobacter cloacae* by disrupting bacterial cell membrane integrity and altering cell surface properties. Targeting these non-lethal mechanisms will lower the risk of bacterial resistance development, highlighting the potential of these herbs and their compounds as natural antibiofilm agents.

## Chemometric analysis

### Multivariate discrimination of extracts based on metabolite profiles

The peak areas of compounds detected by LC-QTOF-MS/MS in -ESI and + ESI modes were subjected to multivariate data analysis to investigate differences among the methanolic extracts of celery, coriander, and parsley. Peak areas of the detected compounds and available metabolite annotations were analyzed using PCA, HCA, and PLS-DA to identify the most discriminant metabolites among the three edible herb extracts and to explore their association with the observed bioactivity.

**Fig. 3:**
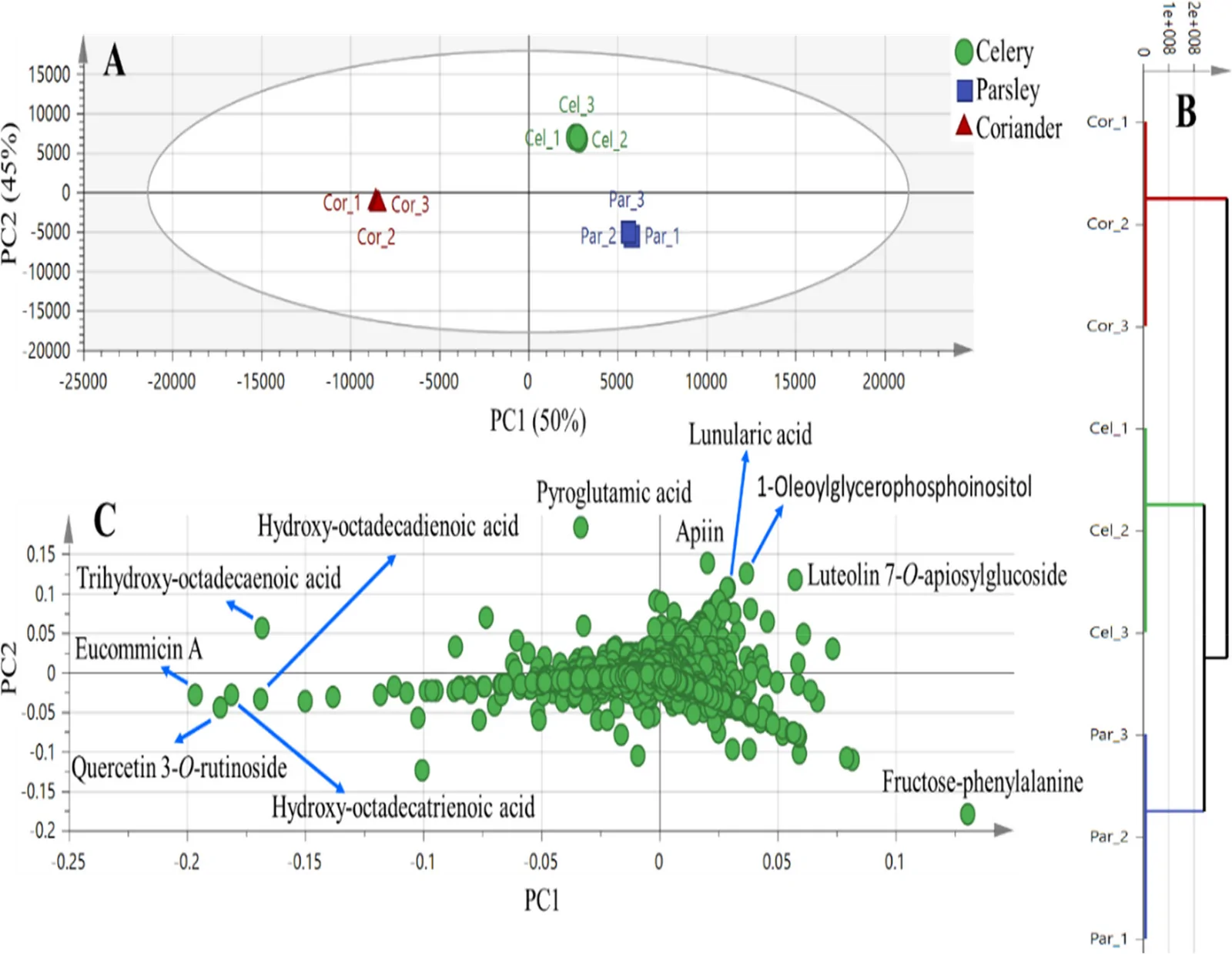
Chemometric analysis of LC-QTOF-MS/MS data obtained in -ESI mode for celery (Cel), coriander (Cor) and parsley (Par) methanolic extracts. **A** PCA scores plot, **B** HCA, and **C** PCA loadings plot

PCA was used for the unsupervised exploration of trends, clustering patterns, and outliers. The PCA scores plot in -ESI mode showed that the first two principal components (PCs) explained 50% (PC1) and 45% (PC2) of the total variance, respectively, accounting for 95% of the overall data variability (Fig. 3A). The analyzed samples were chemically distinct and grouped into three clusters, as further confirmed by HCA (Fig. 3B). As shown in the PCA loadings plot (Fig. 3C), celery samples, located in the positive region of both PC1 and PC2 (+ PC1, +PC2), were characterized by higher abundances of luteolin 7-*O*-apiosylglucoside (12), apiin (28), pyroglutamic acid (106), lunularic acid (123), and 1-oleoylglycerophosphoinositol (131). Coriander samples (-PC1, -PC2, Fig. 3B), showed notable similarity and were characterized by elevated levels of quercetin 3-*O*-rutinoside (8), eucommicin A (167), and C18 fatty acids, including trihydroxy-octadecaenoic acid (121, 124, and 125), hydroxy-octadecatrienoic acid (134), and hydroxy-octadecadienoic acid (140) (Fig. 3C). These metabolites contributed to the clear separation of coriander samples into a distinct cluster, apart from celery and parsley samples (Fig. 3B). Parsley samples (+ PC1, -PC2, Fig. 3B), were mainly characterized by higher abundance of fructose-phenylalanine (108).

After confirming the presence of three distinct classes corresponding to each herb extract, a supervised PLS-DA model with three classes and two latent variables (LVs) was constructed to enhance class separation and identify the most relevant metabolites contributing to discrimination among celery, coriander, and parsley. This was achieved by selecting metabolites with variable importance in projection (VIP) scores greater than 1 (Supplementary Fig. S23), which were consistent with the discriminant metabolites identified in the PCA analysis (Fig. 3).

**Fig. 4:**
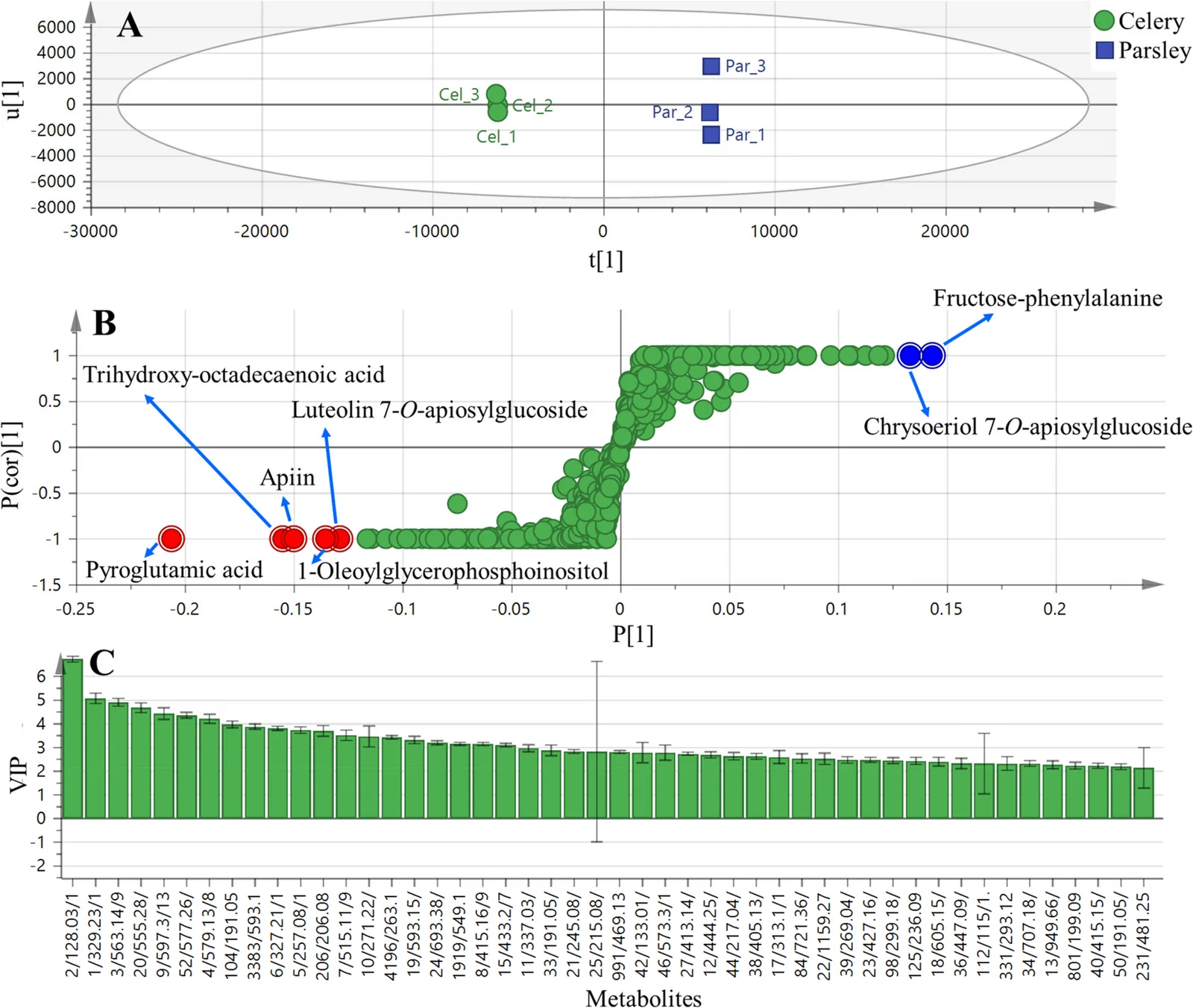
PLS-DA analysis (*p* < 0.005, R^2^ = 99%, Q^2^ = 99%) of the MS dataset in Celery (Cel) against Parsley (Par) extracts in -ESI mode. **A** Scores plot, **B** S-loading plot and **C** VIP scores plot for key metabolites (VIP > 2)

Supervised PLS-DA was further applied to ascertain the possible variability between celery and parsley samples (Fig. 4) where celery and parsley occupied the opposite sides along the two latent variables (Fig. 4A). The model showed high fitting (R^2^ = 0.99) and reproducibility (Q^2^ = 0.99). The markers identified for celery samples were consistent with those observed in the PCA analysis. These metabolites were the ones that had the highest VIP scores (Fig. 4C). Furthermore, the S-plot (Fig. 4B) revealed that both fructose-phenylalanine (108) and chrysoeriol-7-*O*-apiosylglucoside (30) could serve as markers for parsely samples.

**Fig. 5:**
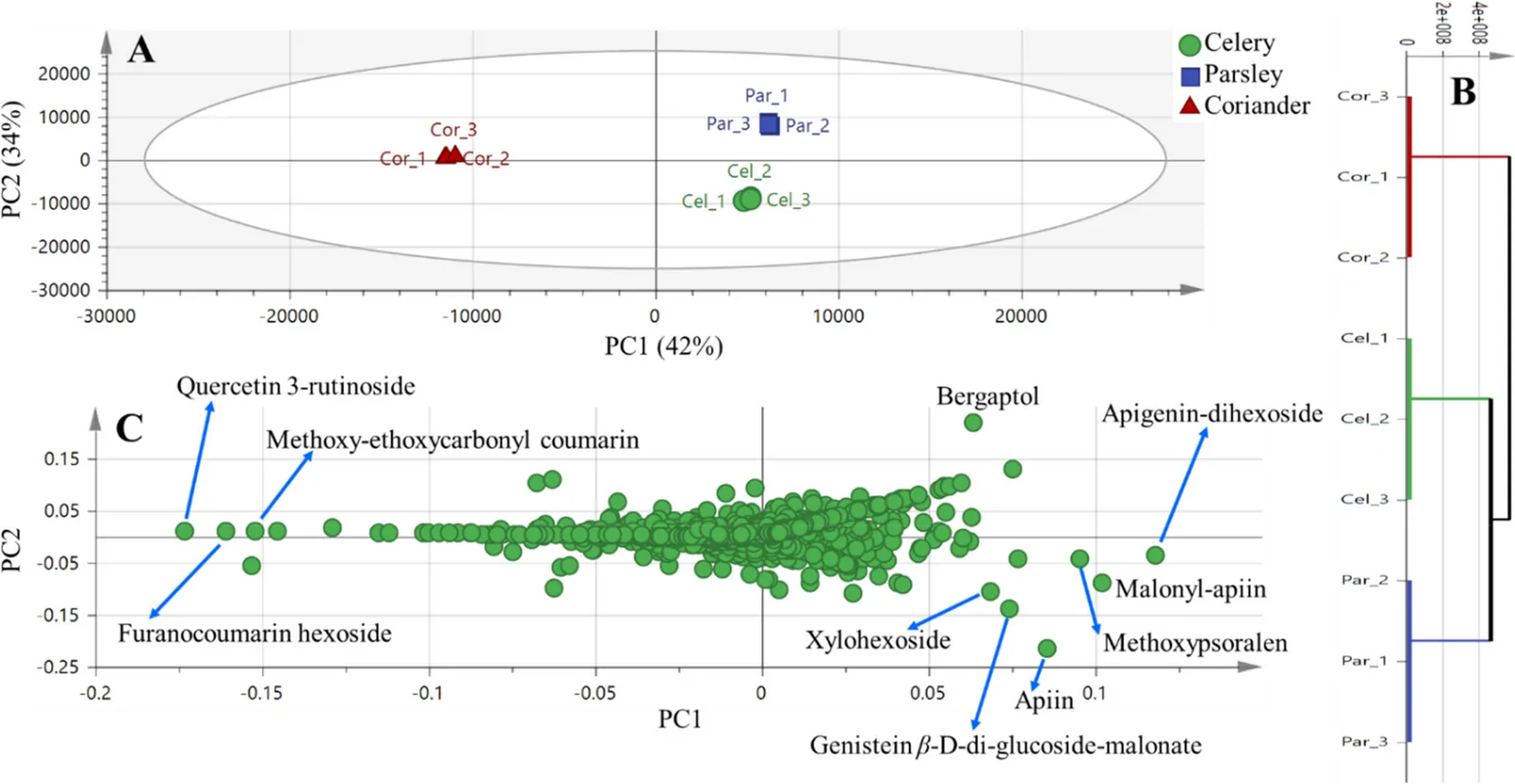
Chemometric analysis of LC-QTOF-MS/MS data obtained in in +ESI for celery (Cel), coriander (Cor) and parsley (Par) extracts. **A** PCA scores plot, **B** HCA, and **C** PCA loadings plot

HCA (Fig. 5B) showed a similar tree to that observed in analysis of -ESI data (Fig. 3B). The loading plot revealed the metabolites responsible for the variability among samples (Fig. 5C). Similar to -ESI data, several metabolites were characteristic for celery, namely, xylohexoside (10), apigenin-dihexoside (26), apiin (28), malonyl-apiin (33), genistein *β*-D-di-glucoside-malonate (37), and methoxypsoralen (94).

Quercetin 3-O-rutinoside (8), furanocoumarin hexoside (isopimpinellin hexoside 82 and coriandrone E hexoside 83), and methoxy-ethoxycarbonyl coumarin (84) were characteristic for coriander samples (Fig. 5C). Parsely samples were distinct from furanocoumarin bergaptol (101).

Chemometric analysis of LC-QTOF-MS/MS data of all samples in + ESI revealed additional markers of interest. First, PCA analysis showed clear clustering into three groups, where coriander was distinctly separated from the other two clusters of celery and parsley (Fig. 5A).

**Fig. 6:**
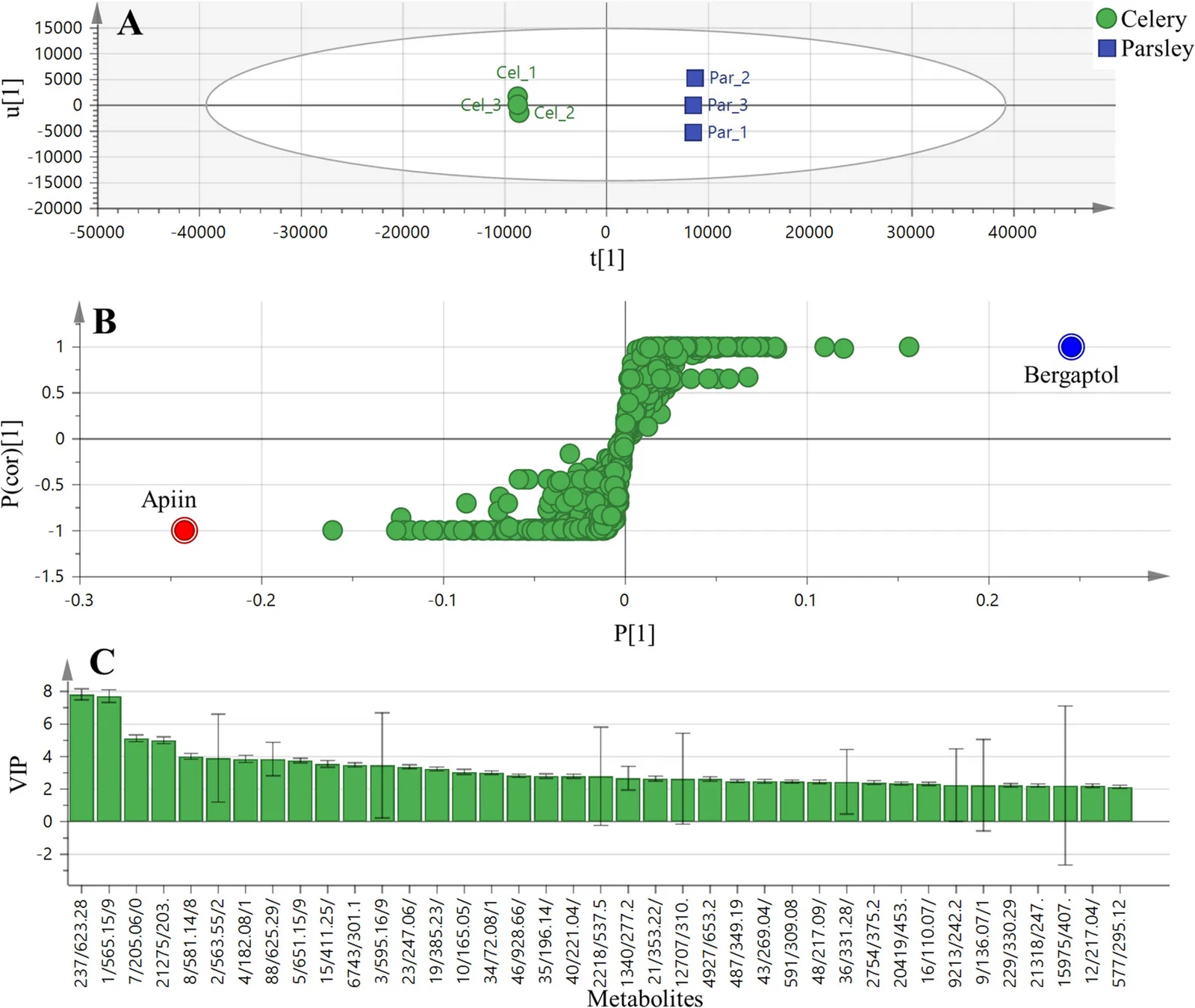
PLS-DA analysis (*p* < 0.01, R^2^ = 99%, Q^2^ = 99%) of the MS dataset in Celery (Cel) against Parsley (Par) extracts in + ESI mode. **A** Scores plot, **B** S-loading plot and **C** VIP scores plot for key metabolites (VIP > 2)

Supervised PLS-DA analysis of all samples in + ESI revealed similar results, yet low R^2^ and Q^2^ values (Fig. S24). It was applied again to reveal more distinction between celery and parsley samples (Fig. 6). The model showed high fitting (R^2^ = 0.99) and (Q^2^ = 0.99) reproducibility values. Apiin (28) and bergaptol (101) could serve as distinct markers for celery and parsley samples, respectively (Fig. 6B). They had the highest VIP scores (Fig. 6C).

### Association of metabolite profiles with antibacterial activity

**Fig. 7:**
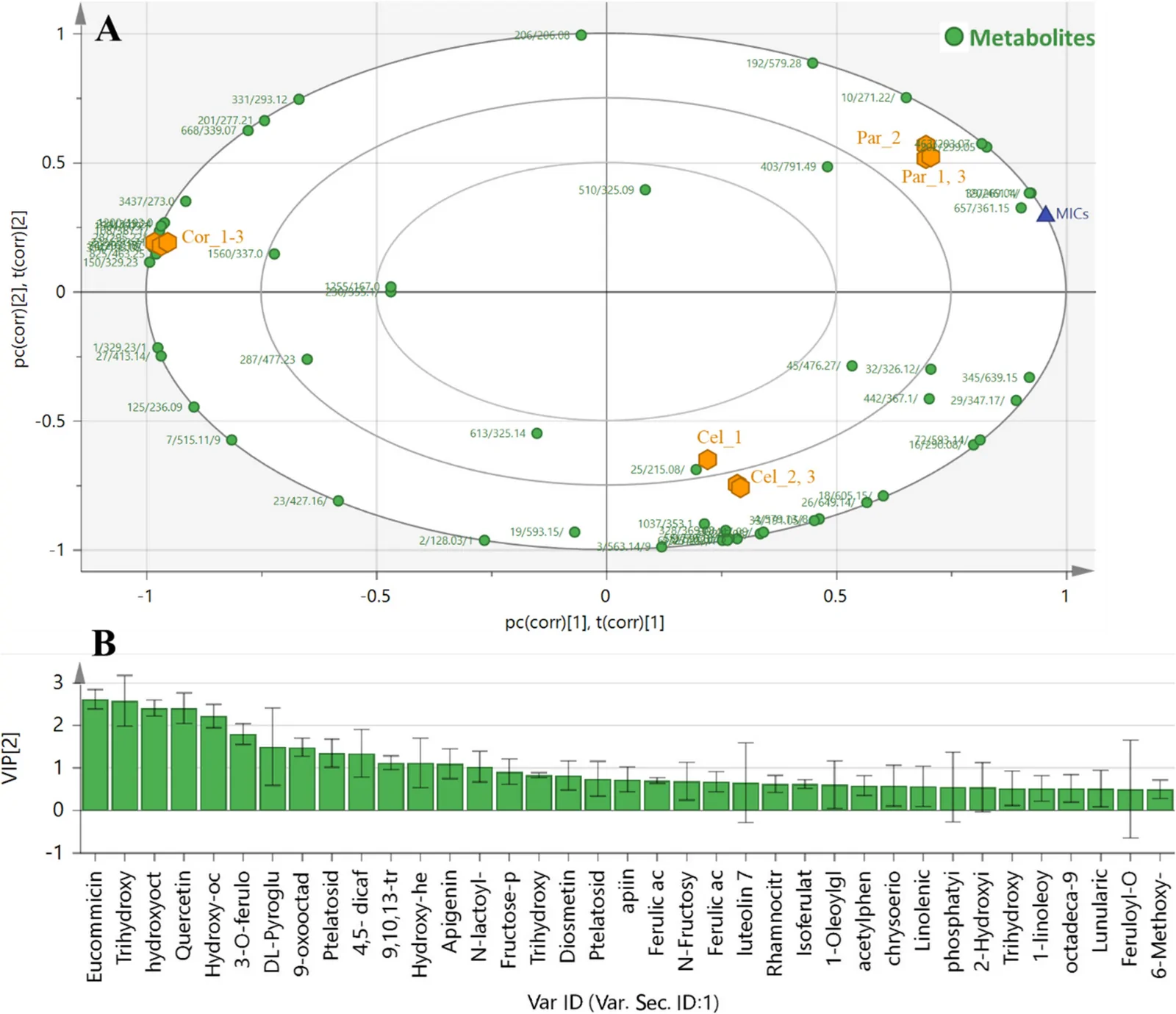
PLS regression analysis (two factors, R^2^ cum = 0.92 and Q^2^ cum = 0.87) between the identified metabolites in celery (Cel), coriander (Cor) and parsley (Par) extracts in -ESI mode and the antimicrobial activity (i.e., MICs). **A** PLS biplot, **B** the key metabolites with VIP ≥ 0.5 were selected

PLS regression (Fig. 7) was conducted to reveal metabolites responsible for the antibacterial activity. The concentration levels of the identified metabolites in -ESI mode of LC-QTOF-MS/MS (variables-X) were modeled against the MIC values of the total extracts (variables-Y). Coriander occupied the far opposite side from MIC values in the PLS biplot which indicates the inverse correlation between the quantification of coriander metabolites and MIC values (Fig. 7A). Parsley was the closest sample to MIC in consistency to their inactivity. The model had high fitting (R^2^ cum = 0.92) and predictive ability (Q^2^ cum = 0.87), the model’s validation parameters were shown in Suppl. Fig. S25. Metabolites with the highest VIP scores were quercetin 3-*O*-rutinoside (8), trihydroxy-octadecaenoic acid (121, 124 and 125), hydroxy-octadecatrienoic acid (134), hydroxy-octadecadienoic acid (140), and eucommicin A (167) (Fig. 7B).

**Fig. 8:**
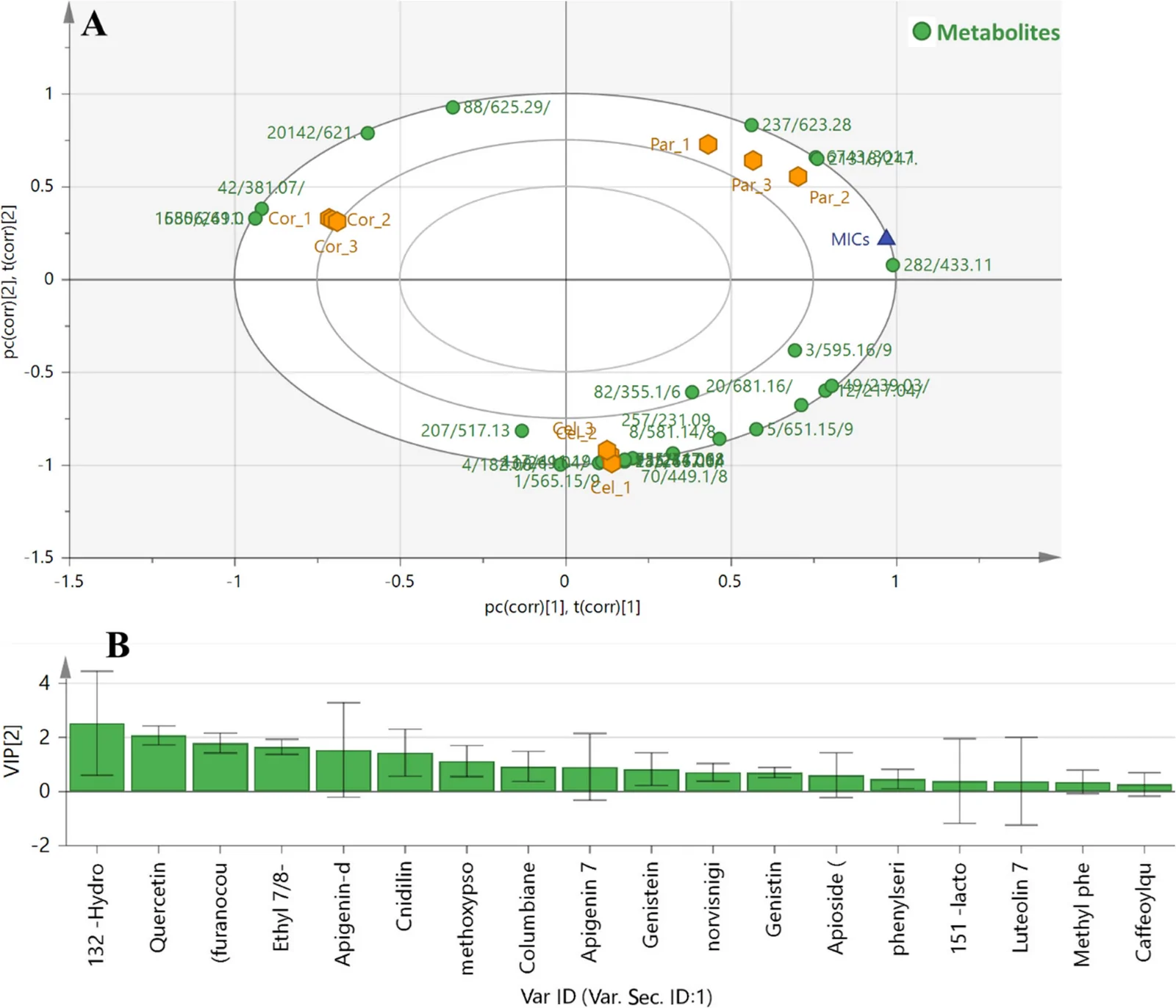
PLS regression analysis (two factors, R^2^ cum = 0.94 and Q^2^ cum = 0.83) between the identified metabolites in celery (Cel), coriander (Cor) and parsley (Par) extracts in + ESI mode and the antimicrobial activity (i.e., MICs). **A** PLS biplot. **B** key metabolites with VIP ≥ 0.3 were selected

Similarly, PLS regression was repeated for metabolites identified in + ESI mode (Fig. 8) where coriander occupied the far opposite side from MIC values in the PLS biplot which indicates the inverse correlation between the quantification of coriander metabolites and MIC values (Fig. 8A). Parsley was the closest sample to MIC in consistency to their inactivity. The model had high fitting (R^2^ cum = 0.94) and predictive ability (Q^2^ cum = 0.83), the model’s validation parameters were shown in Suppl. Fig. S26. Metabolites with the highest VIP scores were quercetin 3-rutinoside (8), furanocoumarin hexoside (isopimpinellin hexoside 82 and coriandrone E hexoside 83), methoxy-ethoxycarbonyl coumarin (84), and 13^2^-hydroxypheophorbide-A methyl ester (166) (Fig. 8B). The metabolites investigated have various mechanisms as antibacterial agents. Quercetin 3-rutinoside, one of the metabolites with a high VIP score, has significantly increased the leakage of proteins, intracellular K+, and AKP by destroying the *E. coli* cell membrane [100]. Similarly, by interfering with bacterial quorum-sensing systems and preventing the formation of biofilms, coumarins and furanocoumarins represented in our study by furanocoumarin hexosides and methoxy-ethoxycarbonyl coumarin may enhance antimicrobial action [114].

## Conclusion

This study provides a comprehensive comparative investigation of celery, coriander, and parsley through the integration of untargeted LC-QTOF-MS/MS metabolomics, chemometric analysis, antibacterial screening against clinical multidrug-resistant uropathogenic *E.coli* isolates, and antibiofilm evaluation. A total of 167 metabolites belonging to diverse phytochemical classes were annotated, revealing distinct metabolite signatures among the three Apiaceae herbs. Chemometric analyses successfully discriminated the extracts and identified key metabolites contributing to their chemical variability and biological activities. Among the investigated extracts, coriander demonstrated the strongest antibacterial activity against multidrug-resistant uropathogenic *E. coli*, while all tested extracts exhibited notable antibiofilm effects. Correlation of metabolomic and bioactivity datasets highlighted hydroxylated fatty acids, quercetin derivatives, coumarins, chlorin-related metabolites, and eucommicin A as potential contributors to the observed activities. Future studies should focus on the experimental validation of the candidate metabolites identified through the integrated metabolomic and bioactivity analyses. The most promising metabolites such as quercetin 3-O-rutinoside, trihydroxy-octadecaenoic acid, hydroxy-octadecatrienoic acid, hydroxy-octadecadienoic acid, and eucommicin A should be isolated, purified, and structurally confirmed using authentic standards and complementary analytical techniques, followed by direct assessment of their antibacterial and antibiofilm activities against MDR uropathogenic *E. coli*. Testing individual metabolites as well as selected combinations would help determine whether the observed effects are attributable to specific compounds or synergistic interactions among multiple constituents. Further mechanistic studies are also required to investigate their effects on bacterial viability and biofilm formation. Finally, evaluation of cytotoxicity and selectivity, followed by validation of the most active candidates in appropriate in-vivo UTI models, would provide stronger evidence for their potential development as anti-UPEC agents. All these studies are necessary to confirm the contribution of the proposed metabolites and to further explore their potential applications in complementary medicine, pharmaceutical development, and food preservation.

## Supplementary Information

Supplementary Material 1.

## Data Availability

All data generated or analysed during this study are included in this article.
