# Supplementary material for: Integrative metabolomics and chemometric analysis reveal metabolites associated with antibacterial and antibiofilm activities of celery, Coriander, and parsley against multidrug-resistant uropathogenic Escherichia coli
Source: BMC Complement Med Ther. 2026 Oct 21;26:313. doi: 10.1186/s12906-026-05610-0 (PMC13685239; doi:10.1186/s12906-026-05610-0)
Supplement: Supplementary file 1 [file 12906_2026_5610_MOESM1_ESM.docx]

**Integrative Metabolomics and Chemometric Analysis Reveal Metabolites Associated with Antibacterial and Antibiofilm Activities of Celery, Coriander, and Parsley Against Multidrug-Resistant Uropathogenic *Escherichia coli***

Shymaa I. A. Abdel-dayem ^1*^, Rania A. El Gedaily^1^, Basma M. Eltanany^2^, Nevine L. Seiffein^3^, Ghada ali han^4^, Sherif M. Afifi^5^, Dalia E. Ali ^6^

^1^ Pharmacognosy Department, Faculty of Pharmacy, Cairo University, 11562 Cairo, Egypt.

^2^ Department of Pharmaceutical Analytical Chemistry, Faculty of Pharmacy, Cairo University, 11562 Cairo, Egyp ^3^Microbiology and Immunology Department Faculty of Pharmacy, Pharos University in Alexandria; Canal El Mahmoudia Street, Beside Green Plaza Complex 21648, Alexandria, Egypt.

^4^ Alexandria Main University Hospital, Alexandria University, 21526 Alexandria, Egypt

^5^ LAQV-REQUIMTE, Laboratory of Bromatology and Hydrology, Faculty of Pharmacy, University of Porto, Rua Jorge de Viterbo Ferreira 228, Porto 4050-313, Portugal

^6^ Pharmacognosy and Natural Products Department, Faculty of Pharmacy, Pharos University in Alexandria; Canal El Mahmoudia Street, Beside Green Plaza Complex 21648, Alexandria, Egypt.

********Corresponding author:*Shymaa I. A. Abdel-dayem (Shymaa.aly@pharma.cu.edu.eg)

**Authors’ e-mail address:**

Shymaa I. A. Abdel-dayem ([shymaa.aly@pharma.cu.edu.eg](mailto:shymaa.aly@pharma.cu.edu.eg)), <http://orcid.org/0000-0001-8152-6781>

Rania A. El Gedaily ([rania.elgedaily@pharma.cu.edu.eg](mailto:rania.elgedaily@pharma.cu.edu.eg))

Basma M. Eltanany (basma.el-tanany@pharma.cu.edu.eg)

Nevine L Seiffein ([Nevine.lotfy@pua.edu.eg](mailto:Nevine.lotfy@pua.edu.eg)), <https://orcid.org/0000-0002-1919-3581>

Ghada ali han ([ghada_han@yahoo.com](mailto:ghada_han@yahoo.com))

Sherif M. Afifi (shshsh38@hotmail.com)

Dalia E. Ali ([Dalia.elsheikh@pua.edu.eg](mailto:Dalia.elsheikh@pua.edu.eg)) h[ttps://orcid.org/0000-0001-5959-733X](https://orcid.org/0000-0001-5959-733X)

**Table S1: Metabolite profiles of methanolic extracts from celery, coriander, and parsley using LC-QTOF-MS/MS in both negative and positive ESI modes.**

| **Peak no.** | **Rt**  **(min)** | **Detected Molecular ion (M-H)^-^/(M/H) ^+^** | **Molecular formula** | **Fragments**  **MS/MS (m/z)** | **Identification** | **Celery** | **Coriander** | **Parsley** | Reference |
| --- | --- | --- | --- | --- | --- | --- | --- | --- | --- |
| **Flavonoids and Flavonoid glycosides** | | | | | | | | |  |
| 1 | 7.59 | 641.1706 | C_28_H_33_O_17_**^+^** | 318/**317**/302/285/97/85 | Rhamnetin 3-Sophoroside | - | - | + | [1] |
| 2 | 7.59 | 639.1553 | C_28_H_32_O_17_**^-^** | 477/**315/**314**/**300/71/241/214 | Isorhamnetin dihexoside | - | - | + | [1] |
| 3 | 7.64 | 593.1499 | C_27_H_29_O_15_**^-^** | 395/**383**/365/353/325/297/279/135 | Vicenin | - | + | - | [2] |
| 4 | 8.06 | 641.1406 | C_28_H_33_O_17_**^+^** | 318/**317**/302/285/97/85 | Rhamnetin mannosyl alloside | - | - | + | [1] |
| 5 | 8.06 | 507.1103 | C_23_H_23_O_13_**^+^** | **303**/85/71 | Quercetin- (acetyl- hexoside) | - | + | - | [3] |
| 6 | 8.26 | 435.1103 | C_21_H_20_O_11_**^+^** | **303/**257/97/85/71 | Quercetin pentoside | - | + | - | [4] |
| 7 | 8.48 | 303.0500 | C_15_H_11_O_7_**^+^** | **303/**257/97/85/71 | Quercetin | - | + | - | [5] |
| 8 | 8.49 | 611.1607 | C_27_H_31_O_16_**^+^** | **303/**257/97/85/71 | Quercetin 3-rutinoside | - | + | - | [1] |
| 9 | 8.54 | 609.1455 | C_27_H_29_O_16_**^-^** | 301/**300/**271/255/243/151 | Quercetin 3-O-rutinosid | - | + | - | [6] |
| 10 | 8.59 | 581.1494 | C_26_H_29_O_15_**^+^** | **287/**153/119/97 | Luteolin 7-Sambubioside (xylohexoside) | + | - | + | [7] |
| 11 | 8.65 | 579.1349 | C_26_H_27_O_15_**^-^** | 447/327**/285/**284/256 | Luteolin 7-arabinopyranosyl-(1->6)-glucoside | + | - | + |  |
| 12 | 8.65 | 579.1349 | C_26_H_27_O_15_**^-^** | 447/**285**/256/133 | luteolin 7-O-apiosylglucoside | + | - | + | [8] |
| 13 | 8.67 | 479.0819 | C_21_H_19_O_13_**^+^** | **303/**257/97/85/71 | Quercetin Glucuronide | - | + | - | [1] |
| 14 | 8.70 | 303.0499 | C_15_H_11_O_7_**^+^** | **303/**257/97/85/71 | Quercetin isomer | - | + | - | [5] |
| 15 | 8.71 | 465.1025 | C_21_H_20_O_12_**^+^** | **303**/257/95/85/71/57 | Quercetin 3-hexoside | - | + | - | [9] |
| 16 | 8.73 | 477.1497 | C_21_H_17_O_13_**^-^** | **301**/283/273/255/178/151 | Quercetin Glucuronide | - | + | - | [10] |
| 17 | 8.76 | 449.1071 | C_21_H_21_O_11_**^+^** | **271** | Apigenin Glucuronide | + | + | - | [1] |
| 18 | 8.78 | 477.0952 | C_21_H_19_O_11_**^-^** | **285**/255/151 | Luteolin-7-O-glucosid | - | + | - | [11] |
| 19 | 8.73 | 477.1497 | C_21_H_17_O_11_**^-^** | 301/ 283/ 255/178/ **151/** 121/107 | Quercetin glucournoide  (Miquelianin) | - | + | - | [1] |
| 20 | 8.78 | 447.0913 | C_21_H_19_O_11_**^-^** | 298/**285**/271/255 | luteolin 5-O-glucoside | + | - | - | [8] |
| 21 | 8.80 | 463.0874 | C_21_H_19_O_12_**^-^** | 301/300/**271**/255 | Hesperetin 7-glucoside | - | + | - | [1, 11] |
| 22 | 8.88 | 595.1649 | C_27_H_31_O_15_**^+^** | **303**/85/71/57 | Quercetin 3,7-dirhamnoside | - | + | - | [12] |
| 23 | 8.91 | 593.1499 | C_27_H_29_O_15_**^-^** | 327/**285**/284/269/255/227 | Kaempferol-3-O-glucorhamnoside | - | + | - | [13] |
| 24 | 9.04 | 565.1547 | C_26_H_29_O_14_**^+^** | **271** | Apioside (apiin) | + | + | + | [1] |
| 25 | 9.08 | 667.1494 | C_29_H_31_O_18_**^+^** | **287**/153/85/69 | Luteolin-7-O-[β-D-apiofuranosyl (1→2) -(6''-O-malonyl)]-β-D-glucopyranoside | + | - | - | [14] |
| 26 | 9.15 | 595.1654 | C_27_H_31_O_15_**^+^** | **271**/153/119/97/87 | Apigenin-dihexoside | + | + | + | [1] |
| 27 | 9.16 | 271.0592 | C_15_H_11_O_5_**^+^** | **271** | apigenin | + | + | + | [1] |
| 28 | 9.20 | 563.1369 | C_26_H_27_O_14_**^-^** | **269**/225 | apiin | + | + | + | [8] |
| 29 | 9.21 | 499.1227 | C_23_H_25_O_11_**^+^** | **303**/257/123/85 | Unknown flavonoid | + | + | - |  |
| 30 | 9.24 | 593.1500 | C_27_H_29_O_15_**^-^** | **299**/284/255 | Chrysoeriol 7-O-apiosylglucoside | + | + | + | [8] |
| 31 | 9.25 | 433.1125 | C_21_H_21_O_10_**^+^** | **271**/153/87/85/69 | Genistin | + | - | + | [9] |
| 32 | 9.40 | 461.1078 | C_22_H_21_O_11_**^-^** | 446/**299**/255/107 | Rhamnocitrin 3-glucoside | + | - | + | [5] |
| 33 | 9.53 | 651.1548 | C_29_H_31_O_17_**^+^** | **271** | Apigenin 7-malonylapiosylglucoside (malonylapiin) | + | - | + | [1] |
| 34 | 9.55 | 649.1403 | C_29_H_29_O_17_**^-^** | 563/**269** | malonylapiin | + | - | - | [8] |
| 35 | 9.56 | 605.1502 | C_28_H_29_O_15_**^-^** | 563/**269** | Apigenin-O-acetyl-hexosyl-pentoside | + | - | + | [15] |
| 36 | 9.57 | 605.1503 | C_30_H_27_O_15_**^-^** | 563/**269**/268/105 | 6’’-acetylapiin | + | - | + |  |
| 37 | 9.61 | 681.1654 | C_30_H_34_O_18_**^+^** | **271**/153/119/97/85 | Genistein β-D-di-glucoside-malonate | + | - | + |  |
| 38 | 9.82 | 739.1364 | C_36_H_35_O_17_**^-^** | 545/445/**269**/193/175/160/149/134 | Apigenin-O-dihexosyl deoxy hexoside | + | - | - | [16] |
| 39 | 11.36 | 271.0600 | C_15_H_11_O_5_**^+^** | **271/**243/145/19 | Apigenin | + | - | + | [1] |
| 40 | 11.43 | 269.0449 | C_15_H_9_O_5_**^-^** | 151/149/**117**/107/65 | Apigenin | + | - | + | [1, 17] |
| 41 | 11.61 | 299.1857 | C_16_H_11_O_6_**^-^** | **284**/256/255/227/151 | Diosmetin | + | + | + | [17] |
| **Isoflavones** | | | | | | | | |  |
| 42 | 11.66 | 255.0629 | C_15_H_11_O_4_**^+^** | 231/**216/**201/174 | Dihydroxyisoflavone | - | + | - |  |
| 43 | 11.82 | 285.0733 | C_16_H_13_O_5_**^+^** | 231/**216** | Isoprunetin | - | + | - | [9] |
| 44 | 12.50 | 269.0420 | C_15_H_9_O_5_**^+^** | 232/**217**/189 | Cumostrol | + | - | + | [9] |
| **Phenolic acids and Phenolic glycosides** | | | | | | | | |  |
| 45 | 1.87 | 315.0714 | C_13_H_15_O_9_**^-^** | 153/152/109/**108** | Protocatechuic acid hexoside (isomer) | - | + | - | [18] |
| 46 | 2.29 | 315.0714 | C_13_H_15_O_9_**^-^** | 153/152/109/**108** | Protocatechuic acid hexoside(isomer) | - | + | - | [18] |
| 47 | 5.04 | 343.1025 | C_15_H_19_O_9_**^-^** | 181/166/**165**/150/108/89/81/59 | Homovanillyl-O-hexoside | - | + | - | [16] |
| 48 | 5.58 | 369.0455 | C_15_H_13_O_11_**^-^** | 127/99/83/**73**/53 | Caffeoylhydroxycitric acid | - | + | - |  |
| 49 | 6.78 | 325.0920 | C_15_H_17_O_8_**^-^** | **119** /117/101 | Coumaric acid-O-hexoside | - | - | + | [19] |
| 50 | 6.92 | 355.1021 | C_16_H_19_O_9_**^+^** | **163**/145/135/117 | Caffeoylquinic acid | + | + | + | [16] |
| 51 | 6.92 | 325.0919 | C_15_H_17_O_8_**^-^** | **325/**163/119/93 | 4-O-beta-D-glucosyl-4-coumarate | - | + | - | [6] |
| 52 | 7.03 | 191.0556 | C_7_H_11_O_6_**^-^** | 171/127/**85** | Quinic acid | + | + | + |  |
| 53 | 7.04 | 353.0870 | C_16_H_17_O_9_**^-^** | 205/ **191**/179 | 5- Caffeolquinic acid | + | + | + | [8] |
| 54 | 7.50 | 411.1622 | C_20_H_27_O_9_**^+^** | **411**/231/205/163/145/135/117 | Chlorogenic acid butyl ester | - | + | - |  |
| 55 | 7.56 | 353.0871 | C_16_H_17_O_9_^-^ | **191**/179/173/85 | Chlorogenic acid (isomer) | + | + | - | [18] |
| 56 | 7.56 | 341.1231 | C_16_H_21_O_8_**^-^** | 179/121/**57** | Caffeic acid hexoside | - | + | - | [20] |
| 57 | 7.75 | 355.1024 | C_16_H_19_O_9_**^-^** | **193/**149/107 | Feruloyl-O-hexoside | - | + | - | [6] |
| 58 | 7.84 | 337.0920 | C_16_H_17_O_8_**^-^** | **191**/127/93 | 2-coumaroylquinic acid | + | + | - | [8] |
| 59 | 7.85 | 167.0345 | C_8_H_7_O_4_**^-^** | 152/**108/**91 | Vanillic acid | - | + | - | [21] |
| 60 | 8.13 | 367.1025 | C_17_H_19_O_9_**^-^** | **191**/173/93/85/59 | 3-O-feruloylquinic acid | + | + | - | [6] |
| 61 | 8.17 | 325.0919 | C_15_H_17_O_8_**^-^** | **119** /117/101/91 | Coumaric acid-O-hexoside isomer (melilotoside) | + | + | + |  |
| 62 | 8.20 | 337.0920 | C_16_H_17_O_8_**^-^** | **191**/127 | 2-coumaroylquinic acid isomer | + | + | - | [8] |
| 63 | 8.31 | 367.1026 | C_17_H_19_O_9_**^-^** | **191**/173/93/85/59 | 4-O-feruloylquinic acid isomer | - | + | - | [6] |
| 64 | 8.14 | 341.1233 | C_16_H_21_O_8_**^-^** | 179**/164**/113/59 | Coniferin | - | - | + | [22] |
| 65 | 8.41 | 367.1025 | C_17_H_19_O_9_**^-^** | **191**/173/93/87/85/59 | 5-O-feruloylquinic acid isomer | - | + | - | [18] |
| 66 | 8.62 | 273.0069 | C_10_H_9_O_7_S**^-^** | **193**/175/149/107 | Ferulic acid sulphate | - | + | - |  |
| 67 | 8.76 | 413.1446 | C_19_H_25_O_10_**^-^** | 125/113/99/101/**59** | Ptelatoside A | + | + | - | [16] |
| 68 | 8.93 | 365.1204 | C_16_H_23_O_8_**^+^** | **365**/329/271/203 | Abietin (Coniferin) | - | - | + | [22] |
| 69 | 8.95 | 193.0501 | C_10_H_9_O_4_**^-^** | **134** | Ferulic acid | - | + | - | [21] |
| 70 | 8.96 | 179.0709 | C_10_H_11_O_3_**^-^** | **164**/163/137/119/109 | Coniferol | - | - | + |  |
| 71 | 9.20 | 515.1183 | C_25_H_23_O_12_**^-^** | **191**/179/135 | 3,5- dicaffeolquinic acid | - | + | - | [8, 23] |
| 72 | 9.32 | 517.1334 | C_25_H_25_O_12_**^+^** | 337/182/**163**/149/145/135 | Isochlorogenic acid | - | + | - |  |
| 73 | 9.32 | 427.1601 | C_20_H_27_O_10_**^-^** | 123/99/79/73/71/**59** | Ptelatoside B | + | + | - | [16] |
| 74 | 9.38 | 515.1185 | C_25_H_23_O_12_**^-^** | 353/**191**/179/173/135 | 4,5- dicaffeolquinic acid | + | - | - | [23] |
| 75 | 9.61 | 341.1235 | C_16_H_21_O_8_**^-^** | 179/**164**/137/119 | Caffeoylhexose | - | - | + | [15] |
| 76 | 9.89 | 193.0500 | C_10_H_9_O_4_**^-^** | 161/134/ **133** | Isoferulate | - | + | - | [6] |
| 77 | 10.75 | 471.1770 | C_24_H_23_O_10_**^-^** | 163/145/**119/**117/101 | 1,6-Bis-O-(4-Hydroxycinnamoyl)Glucose | - | - | + |  |
| **Coumarins** | | | | | | | | |  |
| 78 | 6.33 | 339.0712 | C_15_H_15_O_9_**^-^** | **177/** 149/133/105 | Esculetin-O-glucoside (Esculin) | - | + | - | [16] |
| 79 | 6.92 | 163.0390 | C_9_H_7_O_3_**^+^** | 163/145/135/117/**89** | 6-Hydroxycoumarin | + | + | + |  |
| 80 | 7.21 | 179.0339 | C_9_H_7_O_4_**^+^** | 163/145/135**/ 133**/117 | Daphnetin | - | + | - |  |
| 81 | 7.29 | 339.0714 | C_15_H_14_O_9_**^-^** | **177**/149/133/120 | Aesculin (daphnin) | - | + | - | [16] |
| 82 | 7.54 | 409.1828 | C₁₉H₂_1_O₁₀^+^ | **409**/247/229/203/185/171 | Isopimpinellin hexoside | - | + | + |  |
| 83 | 8.23 | 411.1287 | C₁₉H_23_O₁₀^+^ | 249/231/**203**/188/145 | Coriandrone E hexoside | - | + | - |  |
| 84 | 8.24 | 249.0761 | C_13_H_13_O_5_^+^ | 249/231/**203**/188 | Methoxy-ethoxycarbonyl coumarin | - | + | - |  |
| 85 | 9.02 | 469.1343 | C_21_H_25_O_12_**^-^** | 163/125/**119**/91/71/57 | Plumieride | - | - | + | [22] |
| 86 | 10.69 | 147.0441 | C_9_H_7_O_2_**^+^** | **147**/131/119/103/91 | Coumarin | - | - | + |  |
| 87 | 10.94 | 247.0963 | C_14_H_15_O_4_**^+^** | **203/**175/159/147/131/59/55 | Columbianetin | - | - | + | [24] |
| 88 | 12.02 | 279.1226 | C_15_H_19_O_5_**^+^** | 231/**216**/201/174 | Ulopterol |  |  | + | [25] |
| 89 | 13.09 | 203.0707 | C_12_H_11_O_3_**^-^** | 173/**160**/145/132/92 | 6-Methoxy-3-acetyl-2H-chromene | - | - | + |  |
| 90 | 14.64 | 245.0814 | C_14_H_13_O_4_**^-^** | 217/203/188/174/**161**/**160**/132/89 | Marmesin | + | - | - | [16] |
| 91 | 10.55 | 305.1021 | C_16_H_17_O_6_**^+^** | **203**/175/159/147 | Oxypeucedanin hydrate (Aviprin) | - | - | + | [26] |
| 92 | 12.36 | 231.0653 | C_13_H_11_O_4_**^+^** | 231/**216**/ 213/203/201/174 | Visnagin | - | + | + | [16] |
| 93 | 12.50 | 247.0601 | C_13_H_11_O_5_**^+^** | 247/232/**217/**189 | Isopimpinellin | + | - | + | [16] |
| 94 | 12.53 | 217.0497 | C_12_H_9_O_8_^+^ | 217/**202**/189/174/161/131/115 | Methoxypsoralen (bergapten) | + | - | + | [27] |
| 95 | 12.54 | 239.0314 | C_12_H_9_O_4_**^+^** | 161/133/105/95/**57** | Norvisnigin | + | - | + |  |
| 96 | 13.30 | 287.0912 | C_16_H_15_O_5_**^+^** | **203/**175/159/147/131 | Oxypeucedanin | - | - | + | [26] |
| 97 | 15.28 | 301.1070 | C_17_H_17_O_5_**^+^** | 233**/203/**175/159/147/131/59/55 | Phellopterin | + | + | + | [28] |
| 98 | 15.29 | 301.1457 | C_17_H_17_O_5_**^+^** | **203**/175/159/147/131 | Cnidilin | - | - | + | [29] |
| 99 | 15.29 | 233.0445 | C_12_H_9_O_5_**^+^** | **203/**175/159/147/131 | 5-Hydroxyxanthotoxin | - | - | + |  |
| 100 | 15.48 | 293.0784 | C_18_H_13_O_4_**^+^** | **203/**175/159/147/131/59/55 | Pinnatin | - | - | + |  |
| 101 | 15.49 | 203.0340 | C_11_H_17_O_4_**^+^** | 175/159/**147**/131 | bergaptol | - | - | + | [30] |
| 102 | 15.49 | 271.0965 | C_16_H_15_O_4_**^+^** | **203/**175/159/147/131/59/55 | Isoimperatorin | - | - | + | [26] |
| **Amino acids and Amino acid derivatives** | | | | | | | | |  |
| 103 | 1.02 | 116.0708 | C_5_H_10_NO_2_**^+^** | 116/**70** | Prolin | - | + | + |  |
| 104 | 1.40 | 182.0812 | C_9_H_12_NO_3_**^+^** | **136**/120/107/103 | Phenylserine | + | + | + |  |
| 105 | 1.44 | 290.0875 | C_11_H_16_NO_8_**^-^** | 200/180/168**/128/**123/98/84 | N-Fructosyl pyroglutamate | + | + | + | [31]. |
| 106 | 1.44 | 128.0350 | C_5_H_6_NO_3_**^-^** | **128**/82 | DL-Pyroglutamic acid | + | + | + |  |
| 107 | 1.93 | 236.0921 | C_12_H_14_NO_4_**^-^** | 164/147/119/103/**72** | N-lactoyl-phenylalanine | + | + | + | [32] |
| 108 | 1.95 | 326.1238 | C_15_H_20_NO_7_**^-^** | 206/164/**147**/103 | Fructose-phenylalanine | + | + | + | [31]. |
| 109 | 2.09 | 164.0713 | C_9_H_10_NO_2_**^-^** | 147/**103**/77/72 | Phenylalanine | + | + | + | [31, 32] |
| 110 | 4.91 | 205.0972 | C_11_H_13_N_2_O_2_**^+^** | 188/170/146/**118** | L-Tryptophan | + | + | + | [3] |
| 111 | 5.39 | 203.0819 | C_11_H_11_N_2_O_2_**^-^** | 159/142/**116**/74/56 | L-Tryptophan | - | - | + | [3] |
| 112 | 5.51 | 203.0820 | C_11_H_11_N_2_O_2_**^-^** | 159/142/**116**/74/56 | D-Tryptophan | - | - | + | [3] |
| 113 | 7.18 | 215.0821 | C_12_H_11_N_2_O_2_**^-^** | 171/129/116/**92**/86 | Cyclo-methyl-tryptophan | + | + | - |  |
| 114 | 7.25 | 261.0872 | C_13_H_13_N_2_O_4_**^-^** | 132/98/**74/**59 | Acetyl hydroxy tryptophan | - | - | + |  |
| 115 | 8.31 | 206.0817 | C_11_H_12_NO_3_**^-^** | 164/147/103/91/**58** | Acetyl phenylalanine | - | + | + | [32] |
| 116 | 8.41 | 367.1025 | C_17_H_19_O_9_**^-^** | **191**/133/173/93/85 | Methyl chlorogenate | - | + | - | [11] |
| **Fatty acids and lipids** | | | | | | | | |  |
| 117 | 5.79 | 175.0608 | C_7_H_11_O_5_**^-^** | **115**/113/85/59 | Isopropylmalic acid | + | + | - | [16] |
| 118 | 6.61 | 293.1234 | C_12_H_21_O_8_**^-^** | 131/93/85/**59** | 2-Hydroxyisocaproic acid hexoside | - | + | - | [16] |
| 119 | 8.06 | 361.1495 | C_16_H_25_O_9_**^-^** | 199**/155**/137 | Di-hydroxy-di-methyl-octadienoic acid-O-hexoside | - | + | + |  |
| 120 | 11.18 | 327.2171 | C_18_H_31_O_5_**^-^** | 291/239/229/**211**/183/171 | Trihydroxy-octadecadienoic acid (C18:2) | + | + | + | [33] |
| 121 | 11.60 | 329.2328 | C_18_H_33_O_5_**^-^** | 311/229/**211**/183/171 | Trihydroxy-octadecaenoic acid | + | + | + | [16] |
| 122 | 11.76 | 327.2171 | C_18_H_31_O_5_**^-^** | 201/171**/137/**127/59 | Trihydroxy-octadecadienoic acid (C18:2) | - | + | - | [33] |
| 123 | 11.96 | 257.0815 | C_15_H_14_O_4_^-^ | 213/185/171/**106/**93/79 | Lunularic acid | + | - | - |  |
| 124 | 12.24 | 329.2328 | C_18_H_33_O_5_**^-^** | 211/**171**/155/127 | Trihydroxy-octadecaenoic acid | + | + | - | [16] |
| 125 | 12.42 | 329.2328 | C_18_H_33_O_5_**^-^** | **211**/ 201/171/125 | Trihydroxy-octadecaenoic acid | + | + | + | [33] |
| 126 | 12.73 | 309.2053 | C_18_H_29_O_4_**^+^** | 231/216/213/203/174/**57** | Dioxo-octadecadienoic acid | - | + | - | [34] |
| 127 | 12.83 | 343.2482 | C_19_H_35_O_5_**^-^** | 195/193/**171**/137/121 | 9,10,13-Trihydroxynonadec-11-enoic acid | - | + | - |  |
| 128 | 13.46 | 595.2877 | C_27_H_48_O_12_P**^-^** | 315/**279**/241/152/96/78 | Octadecadienoyl-glycero-3-phospho-myoinositol | + | + | + | [35]. |
| 129 | 13.46 | 579.2831 | C_27_H_47_O_11_S**^-^** | 299/225/206/164/**80** | Octadeca-9,12-dienoic acid sulpho mannosylglycerate | - | + | + |  |
| 130 | 13.71 | 309.2064 | C_18_H_29_O_4_**^-^** | **171**/153/137/96 | Dihydroxy-octadecatrienoic acid | - | + | + | [36] |
| 131 | 13.72 | 597.3035 | C_27_H_50_O_12_P**^-^** | 279/243/**152**/78 | 1-Oleoylglycerophosphoinositol | + | - | - | [35]. |
| 132 | 13.98 | 274.2742 | C_16_H_36_NO_2_**^+^** | **274/** 256/106/88/70/57 | Lauryldiethanolamine | + | + | + |  |
| 133 | 14.59 | 311.2220 | C_18_H_31_O_4_**^-^** | **171**/129/57 | Octadecatrienoic acid | - | + | - | [16] |
| 134 | 15.12 | 293.2121 | C_18_H_30_O_3_^-^ | 275/255/211/**171/**121/96/55 | Hydroxy-octadecatrienoic acid | + | + | + | [33] |
| 135 | 15.15 | 311.1679 | C_17_H_27_O_3_S**^-^** | 197/**183**/170/119/79 | Undecylbenzenesulphonic acid | + | + | + | [33, 37-39]. |
| 136 | 15.20 | 293.1783 | C_18_H_29_O_3_**^-^** | 275/203/171**/**121**/96/**79 | Hydroxy-octadecatrienoic acid | - | + | + | [33] |
| 137 | 15.61 | 476.2773 | C_23_H_43_NO_7_P**^-^** | **279**/196/168/140/78 | 1-Linoleoyl-sn-glycero-3-phosphoethanolamine | + | - | + | [37] |
| 138 | 15.80 | 431.2193 | C_21_H_36_O_7_P**^-^** | 277/271/152/96/**78** | Linoleoyl-sn-glycero-3-phosphate | + | + | + |  |
| 139 | 15.90 | 279.2317 | C_18_H_31_O_2_**^+^** | 173/161/109/95/81/**67** | Octadecadienoic acid | + | + | + | [34] |
| 140 | 15.94 | 295.2272 | C_18_H_31_O_3_**^-^** | **277**/171/125 | Hydroxy-octadecadienoic acid (C18:2) | + | + | + | [16, 33] |
| 141 | 15.97 | 325.1835 | C_18_H_29_O_3_S**^-^** | 239/**183**/119/79 | 4-Dodecylbenzenesulfonate | + | + | + | [33, 37-39]. |
| 142 | 16.26 | 433.2352 | C_21_H_38_O_7_P**^-^** | 279/152/96**/78** | Oleoylglycerone phosphate | + | + | + |  |
| 143 | 16.73 | 293.2115 | C_18_H_29_O_3_**^-^** | **149**/125/57 | 9-oxooctadeca-10,12-dienoic acid | + | + | + |  |
| 144 | 16.75 | 347.1708 | C_16_H_27_O_8_**^-^** | **301**/285/217 | (E)-10-(beta-D-Glucopyranosyloxy)-2-decenoic acid | + | + | + |  |
| 145 | 16.75 | 339.1990 | C_19_H_31_O_3_S**^-^** | 225/**183**/119 | 4-(1-ethylundecyl) benzene sulfonic acid | - | + | - | [33, 37-39]. |
| 146 | 18.56 | 277.2167 | C_18_H_29_O_2_**^-^** | 277/**198**/127/ 109/71 | Linolenic acid (C18:3) | - | + | + | [33] |
| 147 | 18.61 | 271.2274 | C_16_H_31_O_3_**^-^** | 253/**225**/223/55 | Hydroxy-hexadecanoic acid (C16:0) | + | + | + | [40] |
| 148 | 19.31 | 280.2636 | C_18_H_34_NO**^+^** | 161/147/133/109/95/81/69**/55** | Octadecadienoic acid amide | + | + | + | [34] |
| 149 | 19.74 | 732.5605 | C_40_H_79_NO_8_P**^+^** | 109/95/**81**/69/55 | Phosphatidylethanolamine (15:0/20:1) | + | + | + |  |
| 150 | 19.95 | 791.4930 | C_44_H_72_O_10_P**^-^** | **277/**249 | phosphatyidal glycerol (16:1/22:6) or(183n:204n6) | - | - | + |  |
| 151 | 21.26 | 282.2800 | C_18_H_36_NO**^+^** | 240/135/121/114/79/83/**69/**57/55 | Octadecenoic acid amide (oleamide) | + | + | + | [34] |
| 152 | 21.27 | 256.2635 | C_16_H_34_NO**^+^** | 135/121/114/79/83/**69/**57/55 | Hexadecanoic acid amide (palmitamide) | + | + | + | [34] |
| 153 | 21.67 | 338.3416 | C_22_H_44_NO**^+^** | 135/121/114/79/83/**69/**57/55 | Docosenoic acid amide (docosenamide) | + | + | + | [34] |
| 154 | 21.95 | 815.4964 | C_43_H_75_O_12_S**^-^** | **815**/537/225/206/164/112/ 94/80 | 18:3.16:0 sulfoglycolipid | - | - | + |  |
| **Chlorins** | | | | | | | | |  |
| 155 | 19.59 | 885.5502 | C_55_H_73_N_4_O_6_**^+^** | **593/533/**505/460/447 | Pheophytin-B | + | + | + | [41] |
| 156 | 19.60 | 625.3012 | C_36_H_41_N_4_O_6_**^+^** | **593/**533/505/460 | 15^1^ -Lactone-pheophorbide- A | + | + | + | [42] |
| 157 | 19.74 | 637.2645 | C_34_H_38_N_4_O_7_**^+^**+Na | **593/533/**505/460/447 | Bacteriochlorin- A | + | + | + | Arrizabalaga-Larrañaga et al., 2019) |
| 158 | 19.81 | 609.2705 | C_35_H_37_N_4_O_6_**^+^** | **593/533**/505/460 | 13^2^-Hydroxy-pheophorbide- A | + | + | + | [43] |
| 159 | 20.00 | 885.5500 | C_55_H_73_N_4_O_6_**^+^** | 593/533/505/460/447 | Pheophytin-B | + | + | + | [42] |
| 160 | 20.09 | 901.5457 | C_55_H_73_N_4_O_7_**^+^** | **593/533/**505/460/447 | 13^2^ -Hydroxy-pheophytin-B | + | + | + | [44]. |
| 161 | 20.23 | 593.2761 | C_35_H_37_N_4_O_5_**^+^** | 593/533/505/460 | Pheophorbide- A | + | + | + | [45] |
| 162 | 20.35 | 667.2751 | C_37_H_39_N_4_O_8_**^+^** | **593/533/**505/460/447 | 15^2^ -Methoxylactone pheophorbide-B methyl ester | + | + | + | [44] |
| 163 | 20.46 | 621.2698 | C_36_H_37_N_4_O_6_**^+^** | **593/533/**505/460/447 | Methyl pheophorbide b | + | + | + | [44]. |
| 164 | 20.65 | 639.2805 | C_36_H_39_N_4_O_7_**^+^** | **593/533**/505/460 | 15^2^ -Hydroxylactone pheophorbide-A methyl ester | + | + | + | [44] |
| 165 | 20.69 | 887.5661 | C_43_H_79_N_6_O_13_**^+^** | **593/533/**505/460/447 | 13^2^ -Hydroxy-pheophytin-A | + | + | + | [44] |
| 166 | 21.47 | 623.2858 | C_36_H_39_N_4_O_6_**^+^** | **593/533/**505/460/447 | 13^2^ -Hydroxypheophorbide-A methyl ester | + | + | + | [44] |
| **Lignans** | | | | | | | | |  |
| 167 | 7.57 | 707.1813 | C_32_H_36_O_18_^-^ | **191**/173/161 | Eucommicin A | + | + | - | [46]. |

***Retention times are an average value for the different species. **MS/MS fragments were a combination of all the fragments detected in the different species. ***(+) is present in species and (-) is absent. ****Base peak fragments are highlighted in bold.**

**Table S2: Primers used in the study, their band sizes and annealing temperatures.**

| **Primer** | **Primers sequence (5'-3')** | **Band Size** | **Annealing Temperature** |
| --- | --- | --- | --- |
| fimH-f | AACAGCGATGATTTCCAGTTTGTGTG | 465 | 65 |
| fimH-r | ATTGCGTACCAGCATTAGCAATGTCC |  |  |
| pap1 | GACGGC TGTACTGCAGGGTGTGGCG | 328 | 65 |
| pap2 | ATATCCTTTCTGCAGGGATGCAATA |  |  |
| sfa1 | CTCCGGAGAACTGGGTGCATCTTAC | 410 | 65 |
| sfa2 | CGGAGGAGTAATTACAAACCTGGCA |  |  |
| afa-F | CGGCTTTTCTGCTGAACTGGCAGGC | 672 | 65 |
| afa-R | CCGTCAGCCCCCACGGCAGACC |  |  |
| hly S | AGATTCTTGGGCATGTATCCT | 556 | 65 |
| hly AS | TTGCTTTGCAGACTGTAGTGT |  |  |
| cnf S | TTATATAGTCGTCAAGATGGA | 693 | 58 |
| cnf AS | CACTAAGCTTTACAATATTGA |  |  |
| gapA-F | GAAATGGGACGAAGTTGGTG | 104 | 60 |
| gapA-R | AACCACTTTCTTCGCACCAG |  |  |

**Figure S1. LC-QTOF-MS base peak chromatograms of the methanolic extracts of celery, coriander and parsley analyzed in -ESI mode. A, Celery; B, Coriander; C, parsley.**

**Figure S2. LC-QTOF-MS base peak chromatograms of the methanolic extracts of celery, coriander and parsley analyzed in +ESI mode. A, Celery B, Coriander C, parsley**

**Figure S3.** MS/MS spectrum of isorhamnetin dihexoside (**2**, Table S1)

**Figure S4.** MS/MS spectrum of vicenin (**3**, Table S1)

**Figure S5.** MS/MS spectrum of quercetin rutinoside (**8**, Table S1)

**Figure S6.** MS/MS spectrum of chrysoeriol 7-*O*-apiosylglucoside (**30**, Table S1)

 **Figure S7.** MS/MS spectrum of apigenin-O-acetyl-hexosyl-pentoside (**35**, Table S1)

**Figure S8.** MS/MS spectrum of homovanillyl-O-hexoside (**47**, Table S1)

**Figure S9.** MS/MS spectrum of caffeoylquinic acid (**50**, Table S1)

**Figure S10.** MS/MS spectrum of 4-O-beta-D-glucosyl-4-coumarate (**51**, Table S1)

**Figure S11.** MS/MS spectrum of chlorogenic acid (**55**, Table S1)

**Figure S12.** MS/MS spectrum of caffeic acid hexoside (**56**, Table S1)

**Figure S13.** MS/MS spectrum of feruloyl-O-hexoside (**57**, Table S1)

**Figure S14.** MS/MS spectrum of 3-O-feruloylquinic acid (**60**, Table S1)

**Figure S15.** MS/MS spectrum of 3,5- dicaffeolquinic acid (**71**, Table S1)

**Figure S16.** MS/MS spectrum of isopimpinellin (**93**, Table S1)

**Figure S17.** MS/MS spectrum of oxypeucedanin hydrate (**91**, Table S1)

**Figure S18.** MS/MS spectrum of oxypeucedanin (**96**, Table S1)

**Figure S19.** MS/MS spectrum of isoimperatorin (**102**, Table S1)

**Figure S20.** MS/MS spectrum of pheophytin-B (**155**, Table S1)

**Figure S21.** MS/MS spectrum of pheophorbide- A (**161**, Table S1)

**Figure S22.** MS/MS spectrum of methyl pheophorbide b (**163**, Table S1)

**Fig. S23**: PLS-DA analysis (*p* < 0.001, R^2^ = 50 %, Q^2^ = 14 %) of the LC-QTOF-MS data obtained for celery (Cel), coriander (Cor), and parsley (Par) methanolic extracts in -ESI mode A) Scores plot, B) loadings plot, and C) VIP scores plot for key metabolites (VIP > 2.3).

**Fig. S24**: PLS-DA analysis (*p* < 0.001, R^2^ = 50 %, Q^2^ = 11 %) of the LC-QTOF-MS dataset obtained for celery (Cel), coriander (Cor) and parsley (Par) methanolic extracts in +ESI mode. A) Scores plot, B) loadings plot, and C) VIP scores plot for key metabolites (VIP > 2.2).

**Fig. S25**: A) PLS optimization and validation parameters for the antimicrobial effect and metabolites in celery (Cel), coriander (Cor) and parsley (Par) extracts detected in -ESI mode. Bar chart displayed the R^2^_Y_ and Q^2^ as function of number of latent variables. Table displayed the cross-validation CV-ANOVA for model statistical significance. B) Permutation test for the antimicrobial effect displaying the intercept values for R^2^ and Q^2^ in the upper chart. The lower chart showed the relationship between observed vs predicted tests.

**Fig. S26**: A) PLS optimization and validation parameters for the antimicrobial effect and metabolites in celery (Cel), coriander (Cor), and parsley (Par) extracts in +ESI mode. Bar chart displayed the R^2^_Y_ and Q^2^ as function of number of PCs. Table displayed the cross-validation CV-ANOVA for model statistical significance. B) Permutation test for the antimicrobial effect displaying the intercept values for R^2^ and Q^2^ in the upper chart. The lower chart showed the relationship between observed vs predicted test.

1.	Slimestad, R., T. Fossen, and C. Brede, (2020).Flavonoids and other phenolics in herbs commonly used in Norwegian commercial kitchens*.* Food chemistry. **309**: p. 125678.

2.	Wang, Y., et al., (2019).Identification of C-glycosyl flavones by high performance liquid chromatography electrospray ionization mass spectrometry and quantification of five main C-glycosyl flavones in Flickingeria fimbriata*.* BMC chemistry. **13**(1): p. 94.

3.	Vitalini, S., et al., (2021).Phytotoxicity, nematicidal activity and chemical constituents of Peucedanum ostruthium (L.) WDJ Koch (Apiaceae)*.* Industrial Crops and Products. **166**: p. 113499.

4.	Jaiswal, R., et al., (2013).Identification of phenolic compounds in plum fruits (Prunus salicina L. and Prunus domestica L.) by high-performance liquid chromatography/tandem mass spectrometry and characterization of varieties by quantitative phenolic fingerprints*.* Journal of agricultural and food chemistry. **61**(49): p. 12020-12031.

5.	Chernonosov, A.A., E.A. Karpova, and E.M. Lyakh, (2017).Identification of phenolic compounds in Myricaria bracteata leaves by high-performance liquid chromatography with a diode array detector and liquid chromatography with tandem mass spectrometry*.* Revista Brasileira de Farmacognosia. **27**(5): p. 576-579.

6.	Yu, Y., et al., (2023).Inhibitory effect of coriander (Coriandrum sativum L.) extract marinades on the formation of polycyclic aromatic hydrocarbons in roasted duck wings*.* Food Science and Human Wellness. **12**(4): p. 1128-1135.

7.	Bafor, E.E., et al., (2021).Thyme (Thymus vulgaris [Lamiaceae]) Leaves Inhibit Contraction of the Nonpregnant Mouse Uterus*.* Journal of Medicinal Food. **24**(5): p. 541-550.

8.	Lin, L.-Z., S. Lu, and J.M. Harnly, (2007).Detection and quantification of glycosylated flavonoid malonates in celery, Chinese celery, and celery seed by LC-DAD-ESI/MS*.* Journal of Agricultural and Food Chemistry. **55**(4): p. 1321-1326.

9.	Ahmed, S.S.T., J. Fahim, and U.R. Abdelmohsen, (2021).Chemical and biological potential of Ammi visnaga (L.) Lam. and Apium graveolens L.: A review (1963-2020)*.* Journal of advanced Biomedical and Pharmaceutical Sciences. **4**(3): p. 160-176. 10.21608/jabps.2021.55949.1115.

10.	Lachowicz, S., et al., (2019).UPLC-PDA-Q/TOF-MS identification of bioactive compounds and on-line UPLC-ABTS assay in Fallopia japonica Houtt and Fallopia sachalinensis (F. Schmidt) leaves and rhizomes grown in Poland*.* European Food Research and Technology. **245**: p. 691-706.

11.	Faraone, I., et al., (2019).Antioxidant, antidiabetic, and anticholinesterase activities and phytochemical profile of Azorella glabra Wedd*.* Plants. **8**(8): p. 265.

12.	Gómez, J.D., et al., (2018).Broad range flavonoid profiling by LC/MS of soybean genotypes contrasting for resistance to Anticarsia gemmatalis (Lepidoptera: Noctuidae)*.* PloS one. **13**(10): p. e0205010.

13.	Barros, L., et al., (2012).Phenolic profiles of in vivo and in vitro grown Coriandrum sativum L*.* Food Chemistry. **132**(2): p. 841-848.

14.	Liu, G., et al., (2017).Isolation, purification, and identification of the main phenolic compounds from leaves of celery (Apium graveolens L. var. dulce Mill./Pers.)*.* Journal of separation science. **40**(2): p. 472-479.

15.	Kręcisz, M., et al., (2023).Evaluation of bioactive compounds, volatile compounds, drying process kinetics and selected physical properties of vacuum impregnation celery dried by different methods*.* Food Chemistry: p. 135490.

16.	Emad, A.M., et al., (2022).Antioxidant, antimicrobial activities and characterization of Polyphenol-enriched extract of egyptian celery (Apium graveolens L., Apiaceae) aerial parts via UPLC/ESI/TOF-MS*.* Molecules. **27**(3): p. 698.

17.	Mitreski, I., et al., (2014).Polyphenols in representative Teucrium species in the flora of R. Macedonia: LC/DAD/ESI-MS n Profile and Content*.* Natural Product Communications. **9**(2): p. 1934578X1400900211.

18.	de la Luz Cádiz-Gurrea, M., et al., (2013).Comprehensive characterization by UHPLC-ESI-Q-TOF-MS from an Eryngium bourgatii extract and their antioxidant and anti-inflammatory activities*.* Food Research International. **50**(1): p. 197-204.

19.	El-Zaeddi, H., et al., (2017).Preharvest treatments with malic, oxalic, and acetylsalicylic acids affect the phenolic composition and antioxidant capacity of coriander, dill and parsley*.* Food Chemistry. **226**: p. 179-186.

20.	Spréa, R.M., et al., (2020).Lovage (Levisticum officinale WDJ Koch) roots: A source of bioactive compounds towards a circular economy*.* Resources. **9**(7): p. 81.

21.	Zhao, L., et al., (2021).Simultaneous determination of 49 amino acids, B vitamins, flavonoids, and phenolic acids in commonly consumed vegetables by ultra-performance liquid chromatography–tandem mass spectrometry*.* Food Chemistry. **344**: p. 128712.

22.	Zhang, P., et al., (2023).Antihypertensive effects of Pleurospermum lindleyanum aqueous extract in spontaneously hypertensive rats*.* Journal of Ethnopharmacology: p. 116261.

23.	Long, H., M. Stander, and B.-E. Van Wyk, (2012).Notes on the occurrence and significance of triterpenoids (asiaticoside and related compounds) and caffeoylquinic acids in Centella species*.* South African journal of botany. **82**: p. 53-59.

24.	Ge, A.H., et al., (2014).Ultra high performance liquid chromatography with photodiode array detector and quadrupole time‐of‐flight tandem mass spectrometry coupled with discriminant analysis to evaluate Angelicae pubescentis radix from different regions*.* Journal of separation science. **37**(18): p. 2523-2534.

25.	Wan, M.-Q., et al., (2020).Systematic analysis of the metabolites of Angelicae Pubescentis Radix by UPLC-Q-TOF-MS combined with metabonomics approaches after oral administration to rats*.* Journal of Pharmaceutical and Biomedical Analysis. **188**: p. 113445.

26.	Beier, R.C., G.W. Ivie, and E.H. Oertli, (1994).Linear furanocoumarins and graveolone from the common herb parsley*.* Phytochemistry. **36**(4): p. 869-872.

27.	Li, J., et al., (2014).Simultaneous determination of osthole, bergapten and isopimpinellin in rat plasma and tissues by liquid chromatography–tandem mass spectrometry*.* Journal of Chromatography B. **970**: p. 77-85.

28.	Kviesis, J., et al., (2019).Evaluation of furanocoumarins from seeds of the wild parsnip (Pastinaca sativa L. sl)*.* Journal of Chromatography B. **1105**: p. 54-66.

29.	Dugrand, A., et al., (2013).Coumarin and furanocoumarin quantitation in citrus peel via ultraperformance liquid chromatography coupled with mass spectrometry (UPLC-MS)*.* Journal of Agricultural and Food Chemistry. **61**(45): p. 10677-10684.

30.	Sontag, G., E. Frank, and A. Nikiforov, (1983).Identification and determination of bergaptol in citrus fruits*.* Fresenius' Zeitschrift für analytische Chemie. **314**: p. 572-576.

31.	Pogoda, E. and P.M. Kuś, (2025).Application of Liquid Chromatography–Mass Spectrometry-Based Untargeted Metabolomics to Reveal Metabolites Related to Antioxidant Activity in Buckwheat Honey*.* Molecules. **30**(10): p. 2198.

32.	Zhang, P., et al., (2019).Revisiting fragmentation reactions of protonated α-amino acids by high-resolution electrospray ionization tandem mass spectrometry with collision-induced dissociation*.* Scientific reports. **9**(1): p. 6453.

33.	Otify, A., et al., (2015).Mechanistic evidence of Passiflora edulis (Passifloraceae) anxiolytic activity in relation to its metabolite fingerprint as revealed via LC-MS and chemometrics*.* Food & function. **6**(12): p. 3807-3817.

34.	Farag, M.A., et al., (2016).Comparative metabolite profiling and fingerprinting of genus Passiflora leaves using a multiplex approach of UPLC-MS and NMR analyzed by chemometric tools*.* Analytical and bioanalytical chemistry. **408**: p. 3125-3143.

35.	Hsu, F.-F. and J. Turk, (2009).Electrospray ionization with low-energy collisionally activated dissociation tandem mass spectrometry of glycerophospholipids: mechanisms of fragmentation and structural characterization*.* Journal of Chromatography B. **877**(26): p. 2673-2695.

36.	Farag, M.A., et al., (2013).Metabolomics driven analysis of artichoke leaf and its commercial products via UHPLC–q-TOF-MS and chemometrics*.* Phytochemistry. **95**: p. 177-187.

37.	Farag, M.A., et al., (2014).Metabolomic fingerprints of 21 date palm fruit varieties from Egypt using UPLC/PDA/ESI–qTOF-MS and GC–MS analyzed by chemometrics*.* Food Research International. **64**: p. 218-226.

38.	Farag, M.A., et al., (2016).Comparative metabolite profiling and fingerprinting of genus Passiflora leaves using a multiplex approach of UPLC-MS and NMR analyzed by chemometric tools*.* Analytical and bioanalytical chemistry. **408**(12): p. 3125-3143.

39.	Gu, Y., et al., (2021).Critical biodegradation process of a widely used surfactant in the water environment: dodecyl benzene sulfonate (DBS)*.* RSC advances. **11**(33): p. 20303-20312.

40.	Rocchetti, G., et al., (2019).UHPLC‐ESI‐QTOF‐MS phenolic profiling and antioxidant capacity of bee pollen from different botanical origin*.* International Journal of Food Science & Technology. **54**(2): p. 335-346.

41.	Arrizabalaga-Larrañaga, A., et al., (2019).Simultaneous analysis of natural pigments and E-141i in olive oils by liquid chromatography–tandem mass spectrometry*.* Analytical and bioanalytical chemistry. **411**: p. 5577-5591.

42.	Viera Alcaide, I., M. Roca, and A. Pérez Gálvez, (2019).Mass Spectrometry of Non-allomerized Chlorophylls a and b Derivatives from Plants*.*

43.	Danton, M. and C.K. Lim, (2004).Identification of monovinyl tripropionic acid porphyrins and metabolites from faeces of patients with hereditary coproporphyria by high‐performance liquid chromatography/electrospray ionization quadrupole time‐of‐flight tandem mass spectrometry*.* Rapid Communications in Mass Spectrometry. **18**(19): p. 2309-2316.

44.	Keely, B. and J. Maxwell, (1990).Fast atom bombardment mass spectrometric and tandem mass spectrometric studies of some functionalized tetrapyrroles derived from chlorophylls a and b*.* Energy & fuels. **4**(6): p. 737-741.

45.	Matsumoto, T., et al., (2021).Identification of pheophorbide a as an inhibitor of receptor for advanced glycation end products in Mallotus japonicus*.* Journal of natural medicines. **75**: p. 675-681.

46.	Fujiwara, A., et al., (2016).Eucommicin A, a β-truxinate lignan from Eucommia ulmoides, is a selective inhibitor of cancer stem cells*.* Phytochemistry. **122**: p. 139-145.
